# Macrophage activation and polarization modify P2X7 receptor secretome influencing the inflammatory process

**DOI:** 10.1038/srep22586

**Published:** 2016-03-03

**Authors:** Carlos de Torre-Minguela, Maria Barberà-Cremades, Ana I. Gómez, Fátima Martín-Sánchez, Pablo Pelegrín

**Affiliations:** 1 Inflammation and Experimental Surgery Unit, CIBERehd, Murcia’s BioHealth Research Institute IMIB-Arrixaca, Clinical University Hospital *Virgen de la Arrixaca*, 30120 Murcia, Spain

## Abstract

The activation of P2X7 receptor (P2X7R) on M1 polarized macrophages induces the assembly of the NLRP3 inflammasome leading to the release of pro-inflammatory cytokines and the establishment of the inflammatory response. However, P2X7R signaling to the NLRP3 inflammasome is uncoupled on M2 macrophages without changes on receptor activation. In this study, we analyzed P2X7R secretome in wild-type and P2X7R-deficient macrophages polarized either to M1 or M2 and proved that proteins released after P2X7R stimulation goes beyond caspase-1 secretome. The characterization of P2X7R-secretome reveals a new function of this receptor through a fine-tuning of protein release. We found that P2X7R stimulation in macrophages is able to release potent anti-inflammatory proteins, such as Annexin A1, independently of their polarization state suggesting for first time a potential role for P2X7R during resolution of the inflammation and not linked to the release of pro-inflammatory cytokines. These results are of prime importance for the development of therapeutics targeting P2X7R.

Macrophages are essential components of the innate immune response which are involved in the process of acute and chronic inflammation, migrating to local areas of injury or infection. Macrophages are not only key to initiate the inflammatory response and perform antimicrobial activities, but also they contribute to the initiation of the repair process and the resolution of the inflammation. This ability to switch from a pro- to anti-inflammatory state depends on the cytokine environment^1^ and introduced the concept of functionally distinct macrophage phenotypes that have been extensively studied in M1 (classical activated) and M2 (alternatively activated) macrophages^2^,^3^.

Activation of P2X7 receptor (P2X7R) by extracellular ATP in M1 macrophages has been widely studied as a physiological trigger of the NLRP3 inflammasome, and extracellular ATP is considered a danger signal released at sites of inflammation and tissue injury^4^. In M1 macrophages, beyond NLRP3-inflammasome induced IL-1β and IL-18 release, P2X7R also controls the release other proteins, including lysosomal proteases (cathepsin B and S) or inflammasome components (ASC, NALP3, caspase-1)^5^,^6^,^7^,^8^,^9^,^10^, suggesting that in M1 macrophages P2X7R couples to a secretome that has been poorly characterized. In addition, we previously reported that changes in the polarization state of macrophages produces a specific uncoupling of P2X7R signalling from the inflammasome-caspase-1 cascade without changes in receptor activity and that the levels of gene expression of this purinergic receptor were increased in M2 macrophages, suggesting a potential role of P2X7R during the resolving phase of the inflammation^11^,^12^. The significant differences observed in the actions of ATP on the secretion of IL-1β in different states of macrophage polarization^11^, leads us to question whether this can be also associated with changes in the composition of the P2X7R associated secretome independently of the activation of the NLRP3 inflammasome and caspase-1.

Therefore, in this study we applied a proteomic approach to explore the changes in secretome upon P2X7R activation in macrophages polarized either to M1 or M2. We found P2X7R secretome wider than caspase-1 secretome, controlling additional unconventional and conventional routes for protein release. Furthermore, P2X7R stimulation in macrophages was coupled to a set of proteins implicated in dampening inflammation, such as annexin A1 (Anxa1).

## Results

### Characterization of P2X7R secretome in classical and alternative macrophages

**Figure 1:**
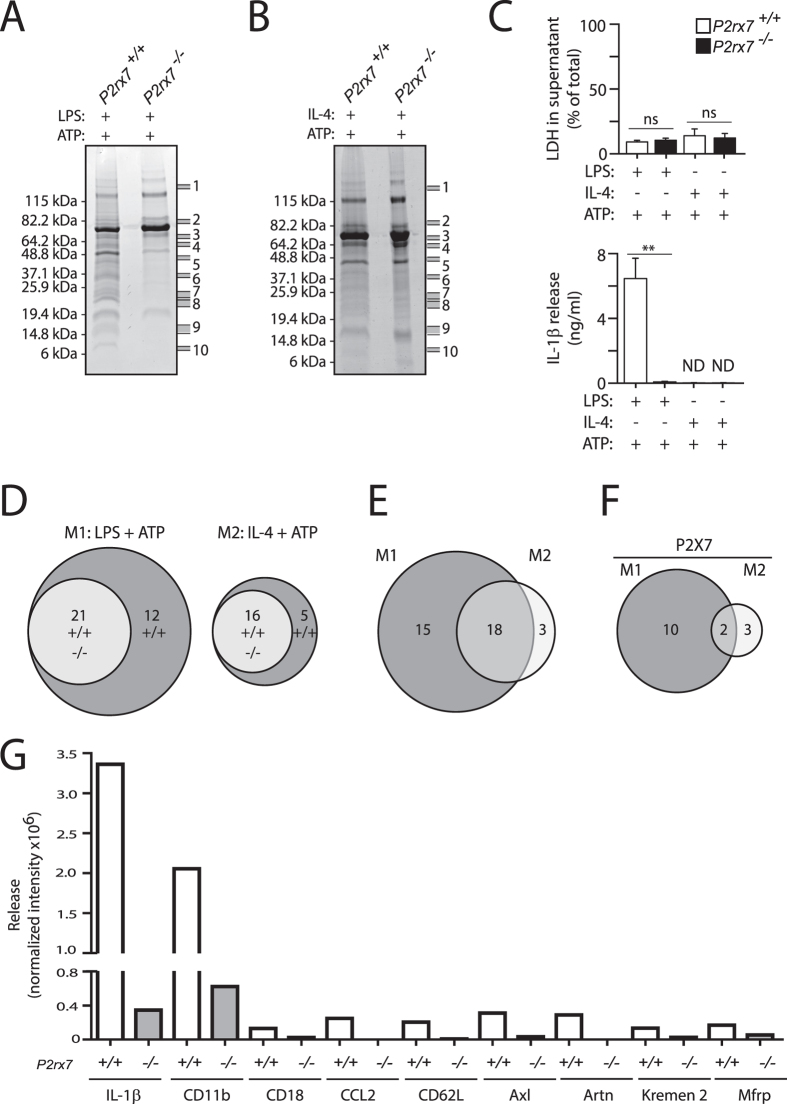
Characterization of P2X7R-dependent secreted proteins from M1 and M2 macrophages. (**A**,**B**) Commassie blue stained SDS-PAGE analysis of proteins secreted upon P2X7R activation of cell-free supernatants from wild-type (*P2rx7*^+/+^) or *P2rx7*^−/−^ BMDMs primed for 4 h with LPS (1 μg/ml, M1) (**A**) or with IL-4 (20 ng/ml, M2) (**B**), followed by stimulation of P2X7R for 20 min with ATP (3 mM); (**C**) Released LDH and IL-1β from macrophages treated as in (**A,B**). (n = 3); **p < 0.01; *ns*, *p* > 0.05 (Student’s *t*-test). (**D**–**F**) Venn diagram showing the relation between the identified proteins secreted by macrophages upon P2X7R activation. (**G**) Antibody array analysis of supernatants from macrophages stimulated as in A using 10 ng/ml LPS. Average quantification of normalized intensity to positive control densities from duplicate positive spots for IL-1β, integrin α-M (CD11b), integrin β-2 (CD18), CCL2, L-selectin (CD62L), tyrosine-protein kinase receptor UFO (Axl), artemin (Artn), kremen protein-2 (Kremen2) and membrane frizzled-related protein (Mfrp). The array was hybridized with a pool of supernatants from *n* = 3 independent experiments. Full arrays are shown in Fig.S1A.

In order to determine P2X7R secretome we used a proteomics methodology to identify the proteins present in cell-free supernatants from wild-type (*P2rx7*^+/+^) or *P2rx7*^−/−^ bone marrow-derived macrophages (BMDMs) polarized either to M1 or M2 and subsequently treated with ATP. The mixture of proteins obtained in these supernatants were fractionated using one dimension gel electrophoresis and 10 bands were selected for LC-MS/MS analysis based in their higher intensity in *P2rx7*^+/+^ supernatant compared with *P2rx7*^−/−^ supernatant (Fig. 1A,B). After mass spectrometry analysis, it was only considered a positive detection when a protein was identified in at least two of the three independent experiments performed.

A prolonged stimulation of P2X7R could be lethal for macrophages^13^ and trigger the unspecific release of their cellular content to the supernatant independently of the potential mechanisms activated by this receptor at short times. Measurements of lactate dehydrogenase (LDH) in supernatants as a marker of cell death associated to the leakage of cytosolic proteins showed a moderately low and similar level of cell death in wild-type and *P2rx7*^−/−^ macrophage supernatant (Fig. 1C). As a positive control we detected the presence of pro-inflammatory IL-1β in the supernatants of wild-type macrophages primed with LPS and treated with ATP, but not in ATP treated *P2rx7*^−/−^ macrophage supernatants (Fig. 1C).

A total of 33 proteins were detected in samples obtained from cell-free supernatants of ATP-treated M1 macro-phages (Table 1 and Supplementary Table S1), while 21 proteins were identified in cell-free supernatants of ATP-treated M2 macrophages (Table 2 and Supplementary Table S2). Interestingly, we observed that some of these secreted proteins were dependent on P2X7R activation and their number were different depending upon macrophage polarization (Fig. 1D): 12 proteins in M1 macrophages and 5 proteins in M2 macrophages. A comparison between the M1 and M2 secretome revealed an important number of common proteins and several proteins whose release is associated with the activation state of the macrophage: 15 proteins were secreted after M1 polarization while only 3 were secreted after M2 polarization (Fig. 1E). Furthermore, 10 of these 15 proteins were dependent on P2X7R activation in M1 macrophages (Fig. 1F) meanwhile 3 proteins were released in a P2X7R dependent manner in M2 macrophage supernatants (Fig. 1F).

The cell-associated function of these 15 proteins secreted upon P2X7R activation is known (Table 1 and Table 2). Interestingly, most of them are known to be secreted via an ER/Golgi-independent pathway: Anxa1 and annexin A2 are known to follow a non-vesicular release pathway^14^,^15^, while high-mobility group box-1 (HMGB1) protein is known to be secreted through a vesicular pathway^16^. We also found secretion of the leaderless proteins thioredoxin (Trx), cystatin B (Cstb) and glutaredoxin-1, those have been previously reported to be released from cells by unconventional mechanisms^17^,^18^,^19^. We also identified the release of MMR and CD14 membrane proteins as dependent on P2X7R activation, these proteins harbor a signal peptide and are associated to the plasma membrane and their detection in the supernatant could be due to a shedding process^20^,^21^.

Although we found the release of novel proteins associated to P2X7R activation in M1 macrophages using LC-MS/MS, this technique was unable to detect the release of the canonical cytokine IL-1β, albeit we could detect it by ELISA (Fig. 1C). To detect the potential release of cytokines dependent upon P2X7R activation, we incubated supernatants from wild-type and *P2rx7*^−/−^ M1 macrophages treated with ATP with a 308-cytokine array membrane (Supplementary Fig. S1A). For these experiments we used a minor LPS dose for priming (10 ng/ml), since most of the target cytokines of the array are strongly upregulated by LPS and this could hide possible fine-tuning release induced by P2X7R activation. The use of a smaller dose of LPS was enough to detect IL-1β release upon ATP stimulation and did not result in an increase of cell death measured by the detection of LDH in supernatants (Supplementary Fig. S1B). This lower LPS concentration was also enough to detect by Western blot the release of P2X7R secretome proteins CD14, CathpB, CstB, identified in the LC-MS/MS (Supplementary Fig. S1C). Using the cytokine array, we found 74 proteins released from wild type M1 macrophages treated with ATP and not present or reduced in *P2rx7*^−/−^ macrophage supernatants (Supplementary Table S3). These included some proteins previously associated to P2X7R activation, such as IL-1β, adhesion molecules or metalloproteases, and proteins identified in our LC-MS/MS experiments such as CD14. From the 74 proteins, 53 were not detected in *P2rx7*^−/−^ macrophage supernatants (Supplementary Table S3). Twenty-three proteins presented a signal >5% of positive spots with a fold change in wild-type respect to *P2rx7*^−/−^ >1.5 (Supplementary Table S3) highlighting 12 with a fold change >3 (Table 3 and Fig. 1G). Interestingly, most of them were proteins localized in the cell membrane with a described function as receptors (Tyrosine-protein kinase receptor UFO, Artemin, Integrin β-2, Integrin α-M, L-selectin, Kremen protein 2 and Membrane frizzled-related protein) suggesting that the release of membrane-associated proteins is one of the main targets of P2X7R activation at short times. P2X7R secretome was mainly composed of proteins with a signal peptide (84%) that followed the classical release pathway (49%) (Supplementary Table S3).

**Figure 2:**
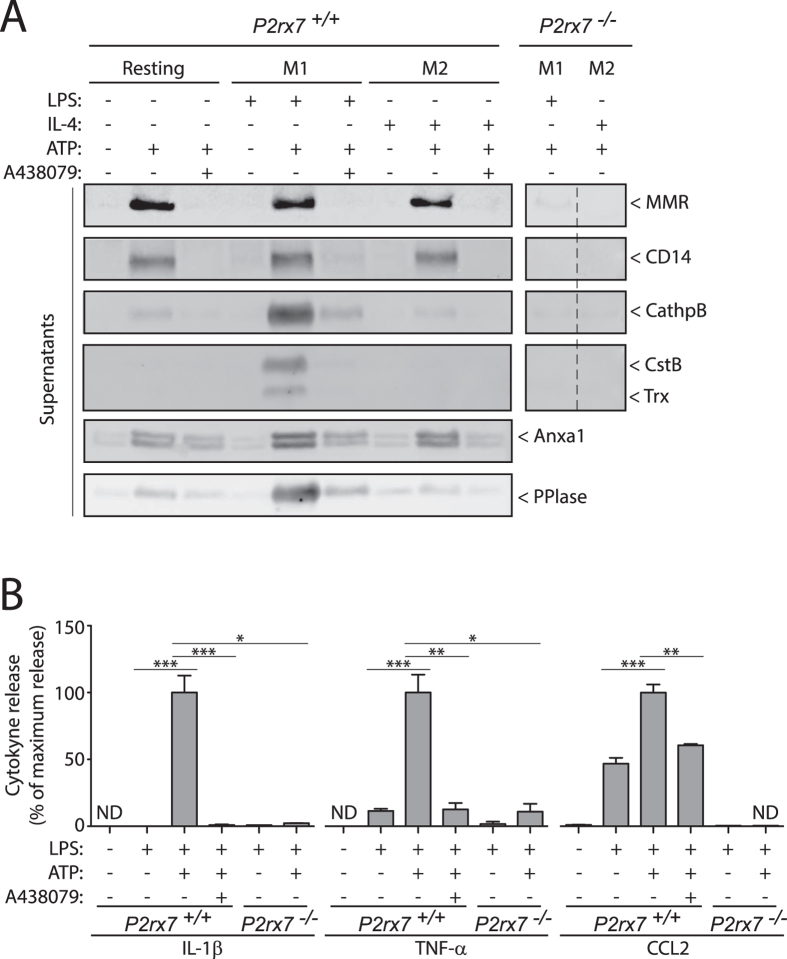
Validation of P2X7R-dependent secretome. (**A**) Western blot analysis of selected P2X7R secretome proteins in cell-free supernatants from wild-type (*P2rx7*^+/+^) or *P2rx7*^−/−^ BMDMs unprimed (resting) (−) or primed (+) for 4 h with LPS (1 μg/ml) (M1) or with IL-4 (20 ng/ml) (M2), followed by no stimulation (−) or stimulation (+) of P2X7R for 20 min with ATP (3 mM); when indicated BMDMs were treated 10 min before and during ATP stimulation with the selective P2X7R antagonist A438079 (25 μM). Control Western blots from cell lysates corresponding to the supernatants could be found in Fig. S2A,B. Western blots lanes for M1 and M2 *P2rx7*^−/−^ BMDMs supernatants is a composited from separated lanes from the same blot (as denoted by a dotted line), full Western blot for *P2rx7*^−/−^ BMDMs supernatants are shown in Fig. S2B and as positive control, they were always run in the same experiment with wild-type BMDMs primed with LPS followed by no stimulation or stimulation with ATP. (**B**) ELISA analysis for released IL-1β, TNF-α and CCL2 from wild-type (*P2rx7*^+/+^) or *P2rx7*^−/−^ BMDMs treated as in (**A**), but primed with 1 μg/ml of LPS for IL-1β or 10 ng/ml of LPS for TNF-α and CCL2 before ATP treatment. Maximum cytokine release detected in LPS + ATP treated macrophages and used for normalization was 13,459.22 pg/ml for IL-1β, 1,188.72 pg/ml for TNF-α and 112.17 pg/ml for CCL2; ND, not detected; Data is presented as mean and s.e.m. of *n* = 2–3 independent experiments for *P2rx7*^+/+^ and n = 1 for *P2rx7*^−/−^ BMDMs. ***p < 0.001; **p < 0.01; *p < 0.05 (one-way ANOVA with Bonferroni’s post-test).

We then selected 9 relevant proteins for the inflammatory process for further validation: MMR, CD14, cathepsin B (CathpB), CstB, Trx, Anxa1, peptidyl-prolyl cis-trans isomerase A (PPIase), TNF-α and C-C motif chemokine 2 (CCL2); and compared their secretion with the control release of IL-1β. By using Western blot and ELISA, despite some targets were initially identified in M2 macrophages, we found that all the 11 selected proteins were able to be released in M1 macrophages upon treatment with ATP and were blocked by using the specific P2X7R antagonist A438079 or *P2rx7*^−/−^ macrophages (Fig. 2A,B and Supplementary Fig. S2A,B). In resting and M2 macrophages, P2X7R induced the release of MMR, CD14 and Anxa1 (Fig. 2A and Supplementary Fig. S2A,B), suggesting that these macrophages could release anti-inflammatory Anxa1 in the absence of pro-inflammatory cytokines. CathpB, CstB, TNF-α and CCL2 were released at baseline and their release was increased upon P2X7R activation (Fig. 2A,B).

### P2X7R controls secretome release at different levels

**Figure 3:**
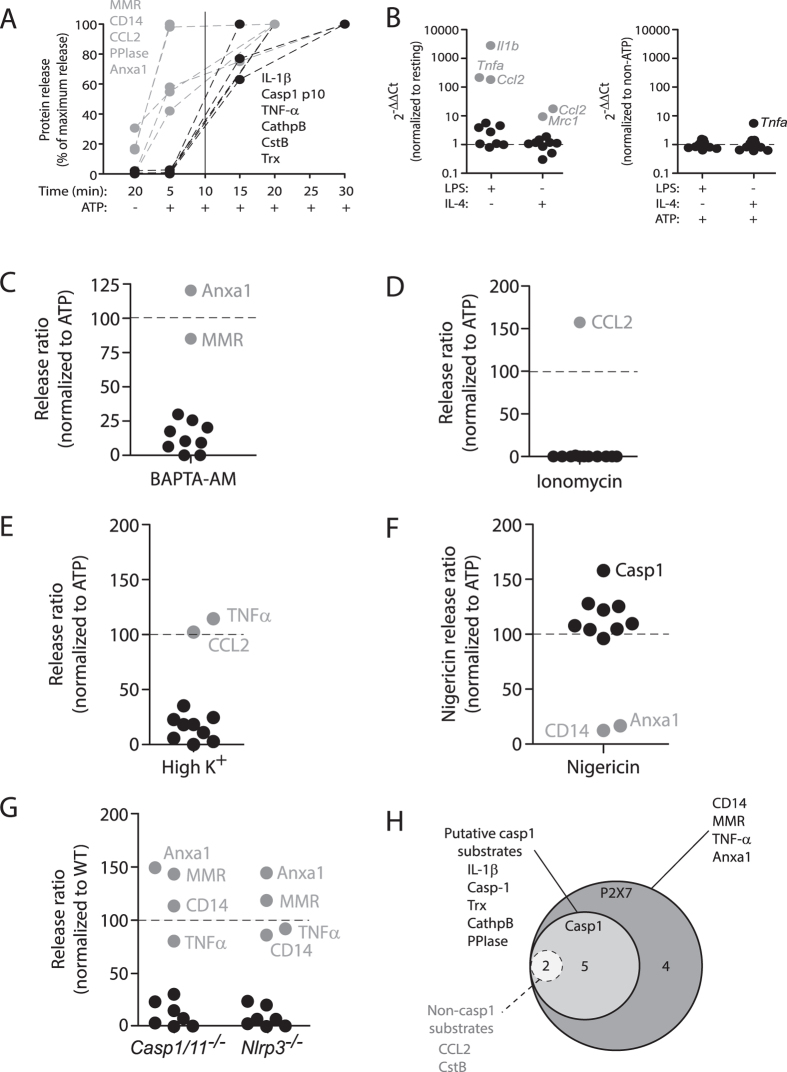
P2X7R secretome do not depend on *de novo* gene transcription and it is wider than caspase-1 secretome. (**A**) Quantification of selected P2X7R secretome proteins in cell-free supernatants from BMDMs primed for 4 h with LPS (10 ng/ml for CCL2 and TNF-α or 1 μg/ml for the other), followed by no stimulation (−) or stimulation (+) of P2X7R with ATP (3 mM) for different times as indicated. (**B**) Relative gene expression for all selected P2X7R secretome proteins determined by quantitative RT-PCR. Left panel show fold change for LPS or IL-4 treatments relative to unprimed (resting) macrophages; Right panel show fold change for LPS + ATP or IL-4 + ATP treatments relative to LPS or IL-4 primed macrophages respectively; relative expression data for each gene is shown in Fig. S4. (**C**,**D**) Quantification of selected P2X7R secretome proteins in cell-free supernatants from BMDMs primed as in (**A**) followed by no stimulation or stimulation for 20 min with ATP (3 mM) (**C**) or Ionomycin (5 μM) (**D**); when indicated BMDMs were treated 10 min before and during ATP stimulation with the cell permeable Ca^2+^-chelator BAPTA-AM (100 μM). Release ratio of ATP in BAPTA-AM treated cells (**C**) or cells treated with ionomycin (**D**) is relative to ATP treatment in physiological buffer. (**E**) Quantification of selected P2X7R secretome proteins in cell-free supernatants from BMDMs primed and stimulated as in normal (2 mM) or high (145 mM) K^+^ buffer. Release ratio of ATP in high K^+^ buffer treated cells is relative to ATP treatment in physiological buffer. (**F**) Relative release ratio of selected P2X7R-dependent secretome proteins induced by nigericin (5 μM) treatment relative to ATP treatment. (**G**) Relative release ratio of selected P2X7R-dependent secretome proteins in *Casp1/11*^−/−^ or *Nlrp3*^−/−^ macrophages relative to wild type macrophages. (**H**) Venn diagram showing the relation between proteins secreted by macrophages upon P2X7R activation and their dependence on caspase-1 activation. Two proteins are dependent on caspase-1, but are not direct substrates of caspase-1 (dotted line circle). Western blots used for densitometry and ELISAs are shown in Fig. S3, S5, S6A and S7.

The secretion of the 11 selected proteins was fast and occurred within 20 min of ATP stimulation (Fig. 2A,B). Careful kinetics assays revealed a set of proteins (MMR, CD14, PPIase, Anxa1 and CCL2) that are early released upon 5 min after P2X7R stimulation (Fig. 3A and Supplementary Fig. S3A–C). We then confirmed that the release of proteins upon P2X7R activation was not dependent on *de novo* gene transcription, since ATP treatment was not increasing gene expression for any of the studied proteins. However, LPS and IL-4 were modulating gene expression as expected while polarizing macrophages to M1 or M2 prior to ATP treatment (Fig. 3B and Supplementary Fig. S4).

P2X7R is a non-specific cationic ion channel, and upon ATP ligation allows the entrance of Ca^2+^ and Na^+^ into the cell and the efflux of K^+^, being intracellular Ca^2+^ rise an important second messenger downstream P2X7R^22^. To study the dependence of intracellular Ca^2+^ elevation on the release of the different P2X7R associated proteins, we studied their release in the presence of the cell permeable Ca^2+^ chelator BAPTA-AM. From the 11 secretome proteins studied, BAPTA-AM was able to impair the release of 9 and did not affect the release of MMR and Anxa1 (Fig. 3C and Supplementary Fig. S5). However, intracellular Ca^2+^ rise was not solely the responsible for P2X7R secretome since elevation of intracellular Ca^2+^ using the Ca^2+^-ionophore ionomycin was able to induce only the release of CCL2, and was unable to induce the release the other 10 proteins analysed (Fig. 3D and Supplementary Fig. S5).

Caspase 1 (Casp-1) controls the unconventional release of different proteins^23^ and since P2X7R is a potent inducer of Casp-1 activation in M1 macrophages via the NLRP3 inflammasome, we then analysed which P2X7R secretome proteins were controlled by Casp-1 activation. Drop of intracellular K^+^ upon P2X7R ion channel opening is an important step for NLRP3 inflammasome assembly and Casp-1 activation, and we found here that impairing K^+^ efflux from macrophages did not affected release of TNF-α and CCL2, but was able to inhibit the release of the other nine analysed proteins (Fig. 3E and Supplementary Fig. S6A). To further analyse the role of Casp-1 activation, we stimulated macrophages with nigericin, a well-established inducer of the NLRP3 inflammasome via K^+^ efflux and independent on P2X7R^24^. When compared to P2X7R stimulation, nigericin was unable to induce the release of CD14 and Anxa1, but induced the release of the other nine analysed proteins (Fig. 3F and Supplementary Fig. S6A). It was surprising to found that TNF-α and CCL2 release was not affected when P2X7R was activated in high extracellular K^+^, but nigericin was able to induce their release. We then found that nigericin was increasing intracellular Ca^2+^ to an extent comparable to P2X7R activation and that blocking K^+^ efflux did not impair intracellular Ca^2+^ rise (Supplementary Fig. S6B). Since both proteins were highly dependent on intracellular Ca^2+^ rise, it is tempting to speculate that nigericin could be controlling their release via elevation of Ca^2+^ and not via K^+^ depletion. The fact that the release of CD14 and Anxa1 was dependent on K^+^ efflux when activating the P2X7R, but nigericin did not induce its release, suggests that P2X7R signalling activates additional pathways than nigericin in macrophages important for the release of these proteins.

We next compared the release of proteins induced by ATP treatment from wild-type macrophages with macrophages deficient on Casp-1 or NLRP3. The release of Anxa1, MMR, CD14 and TNF-α after P2X7R activation was not affected in inflammasome knock-out macrophages (Fig. 3G and Supplementary Fig. S7), suggesting that their release is independent on Casp-1 activation. Therefore P2X7R secretome appears wider than Casp-1 secretome in M1 macrophages, and from the 7 proteins controlled by Casp-1, only 5 were direct substrates of the protease after analysing their sequence using the Granzyme2Applet 1.0 software^25^ (Fig. 3H).

**Figure 4:**
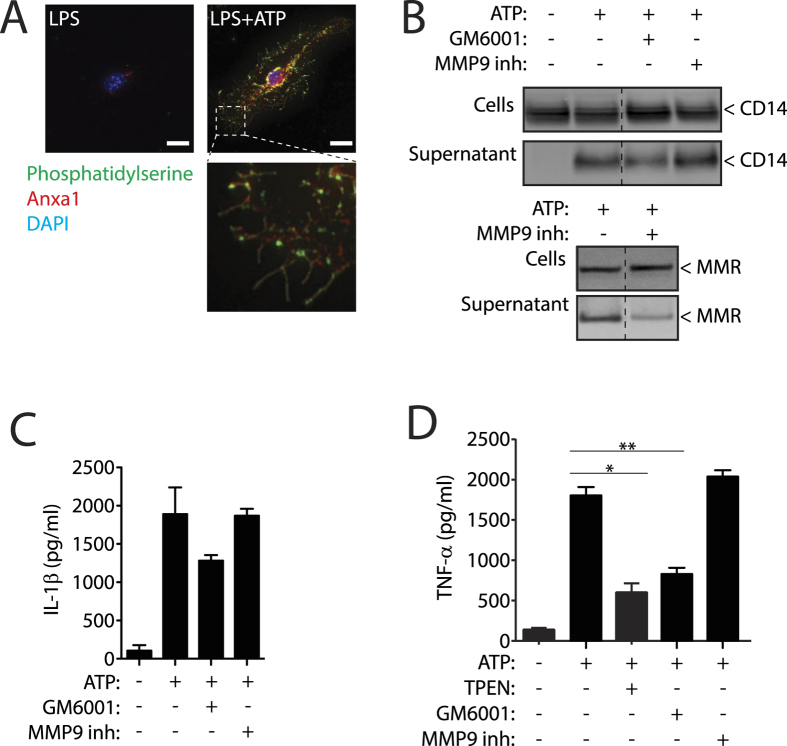
P2X7R controls different mechanisms in Anxa1, CD14, MMR and TNF-**α** release. (**A**) Representative high intensity projection deconvolved image of a mouse bone marrow-derived macrophages (BMDMs) primed for 4 h with LPS (1 μg/ml), followed by stimulation or not for 5 min with ATP (3 mM) and stained for phosphatidylserine with annexin V-FITC (green), for anxa1 (red) and for nuclei (DAPI, blue) without cell permeabilization; scale bar = 10 μM. (**B**) Western blot for CD14 and MMR in cell-free supernatants and cell lysates from BMDM primed as in (**A**) but with 20 min of ATP (3 mM) stimulation; 10 min before and during ATP stimulation cells were incubated with the general metalloprotease inhibitor GM6001 (100 nM) or with the specific MMP9 inhibitor I (100 nM). Western blots are representative of *n* = 2 independent experiments and composite is from lanes of the same membrane. (**C,D**) ELISA analysis for released IL-1β (**C**) or TNF-α (**D**) from BMDMs treated as in (**B**) and additionally 10 min before and during ATP stimulation cells were incubated with the Zn^2+^ chelator TPEN (50 μM). Data is presented as mean and s.e.m. of *n* = 3 independent experiments.

The four proteins released independent on Casp-1 were associated to the plasma membrane and three of them were released both in M1 and M2 macrophages. MMR and TNF-α present a transmembrane domain and are integral membrane proteins, CD14 is anchored to the membrane by a glycosyl-phosphatidylinositol tail, and Anxa1 binds to plasma membrane phospholipids in the presence of Ca^2+^. We found that the flip of phosphatidylserine to the outer plasma membrane leaflet induced upon P2X7R activation^26^ is accompanied by Anxa1 exposure on macrophages after ATP stimulation (Fig. 4A). Membrane Anxa1 staining was lost when Ca^2+^ was chelated with EGTA (Supplementary Fig. S8A), indicating that Ca^2+^ chelation induced the release of Anxa1 from exposed phosphatidylserine, and this could explain the release of Anxa1 in the presence of BAPTA-AM found on Fig. 3C. As controls, we also found staining for Anxa1 and phosphatidylserine in permeabilised resting macrophages or when Casp-1 knock-out macrophages were activated with ATP (Supplementary Fig. S8A), this explains the release of Anxa1 independent of the activation of caspase-1.

It is known that P2X7R activation induces the activation and release of metalloproteases (MMP)^27^, and that MMR and TNF-α could suffer MMP-dependent shedding^20^,^28^. Treatment of macrophages with the general MMP inhibitor GM6001 during ATP stimulation reduced the release of TNF-α (Fig. 4B,D), also affecting slightly the release of CD14 and IL-1β (Fig. 4B,C). Specific inhibition of MMP9 was able to block ATP induced MMR release without affecting the release of CD14, TNF-α or IL-1β (Fig. 4B–D), while general inhibitors of MMP as GM6001 or the Zn^2+^ chelator TPEN completely abrogated P2X7R induced TNF-α release (Fig. 4D).

In summary, our data present the characterization of P2X7R secretome in macrophages and showed that proteins released under P2X7R activation are more abundant than those secreted only by inflammasome stimulation. Furthermore, we found that P2X7R controls different mechanisms involving metalloprotease activation, plasma membrane phosphatidylserine flip, increase of intracellular Ca^2+^ or efflux of K^+^ that regulate either the unconventional and conventional release of the proteins.

## Discussion

Inflammation is an innate immune response coordinated by a complex system of sensors that induces the release of different signalling molecules, including cytokines and other immune mediators^29^. The purinergic P2X7R constitute a key innate immune sensor for elevated concentrations of extracellular ATP, which are present in areas of tissue damage and inflammation. P2X7R activation promotes the assembly of the NLRP3 inflammasome and the unconventional release of the pro-inflammatory leaderless cytokines IL-1β and IL-18^6^. In this study we characterised P2X7R secretome beyond IL-1β and found the release of novel cell-related proteins, including the cell-surface MMR and CD14, the unconventional release of the lysosomal proteins CathpB and CstB, or the redox regulatory proteins Trx and glutharedoxin-1, among others. Also, our study found that P2X7R is able to control the classical release of cytokines such as CCL2 and TNF-α, independent on *de novo* gene transcription. This mechanism is different from a recent work demonstrating TNF-α and CCL2 induction after P2X7R activation in microglia via gene transcription^30^. In fact, any of the proteins detected and analysed in our study was upregulated at gene expression level upon P2X7R activation and most of them have been previously associated to microparticles^31^. It is known that P2X7R activation in macrophages induces the release of microparticles^8^,^32^, suggesting that part of the secretome associated to P2X7R could be associated to microparticle release.

In alternative activated M2 macrophages we found that P2X7R controls the release of a subset of proteins, which included MMR, CD14 and annexin A1, A2 and A4, suggesting a potential role for P2X7R signalling during resolution of the inflammation. Intracellular pathways activated downstream P2X7R where interconnected to induce the release of different proteins, being some of them independent on the activation of caspase-1 by the NLRP3 inflammasome. P2X7R also induces the activation of metalloproteases^27^, a process that we found was coupled to the fast release of membrane receptor MMR after P2X7R stimulation, regardless of macrophage polarization. At plasma membrane level, P2X7R control a fast flip of phosphatidylserine to the outer leaflet of the plasma membrane^26^ and our data suggests that this mechanism could explain the early release of Anxa1 independently of Casp-1 activation and macrophages polarization, as this phospholipid flip has been found important for the release of different annexins^33^. We also observed a quick release proteins secreted by a vesicular pathway, such as CCL2 or PPIase, suggesting that P2X7R activation acts like a fine tuning to accelerate the release of these proteins previously primed with LPS. We found the secretome of P2X7R wider than the secretome controlled by Casp-1, being P2X7R also functional in M2 macrophages, where Casp-1 is not active^11^.

The resolution of the inflammation is a complex, not fully understood, process. There are several programs to resolve inflammation, being one of the initial steps the phagocytosis of apoptotic neutrophils by macrophages, a process that polarize macrophages to alternative M2 phenotypes^34^. In fact, M2 macrophages generated *in vitro* with IL-4 or IL-13 stimulation resemble to pro-resolving macrophages found *in vivo*^35^. M2 macrophages are able to damp pro-inflammatory pathways (i.e. NF-κB) and produce a plethora of anti-inflammatory and pro-resolving molecules, including IL-10 and lipids such as resolvins and maresins^34^. Previous evidences from our lab show that P2X7R, a receptor classified as pro-inflammatory, is functional in M2 macrophages^11^. In fact, P2X7R expression is higher in M2 when compared to M1 macrophages and its activation in M2 macrophages does not couple to the activation of the inflammasome or the production of reactive oxygen species^11^,^12^, however the function of P2X7R in resting and M2 macrophages still unknown. Here we show that P2X7R controls the unconventional release of a subset of proteins in resting and M2 macrophages, including the release of the LPS co-receptor CD14 or Anxa1. Interestingly, CD14 promotes the non-inflammatory recognition and phagocytosis of apoptotic cells as membrane receptor^36^, although the role of soluble CD14 in this mechanism it is not been proved yet^37^. Lack of CD14 from macrophages upon P2X7R activation could also result in a decrease on LPS responsiveness and therefore decreasing the grade of M1 polarization. Anxa1 is a potent anti-inflammatory molecule, being one of the main mediators of the anti-inflammatory effects of corticoids and involved in the phagocytic clearance of apoptotic neutrophils in the inflammatory site^38^,^39^. Together we found P2X7R linked to an anti-inflammatory function in resting and M2 macrophages, without the secretion of pro-inflammatory cytokines. Our data suggest a role for P2X7R in macrophages that could be important for the resolution of the inflammation, controlling P2X7R the unconventional release of proteins beyond the activation of inflammasomes and caspase-1.

## Methods

### Mice

C57 BL/6 (wild type, WT) mice were purchased from Harlan. P2X7R-deficient mice in C57 BL/6 background (*P2rx7*^−/−^)^24^ were purchased from Jackson NLRP3-deficient (*Nlrp3*^−/−^)^40^ and Caspase-1 (*Casp1^−/−^ Casp11*^−/−^)^41^ were in C57 BL/6 background. All animal experiments were done with approval by and in accordance with regulatory guidelines and standards set by the Institutional Animal Care and Use committee of Clinical University Hospital *Virgen de la Arrixaca*.

### Differentiation and *in vitro* stimulation of macrophages from mouse bone marrow precursors

BMDMs were obtained as described^42^ and cells were primed for 4 h at 37 °C with different doses of LPS (as indicated in the figure legends, M1 macrophage polarization) or with 20 ng/ml of IL-4 (M2 macrophage polarization) or left in medium alone to obtain resting macrophages. Cells were then rinsed three times with different buffers (Et or Et-high K^+^, as indicated in the figure legends, if no indication cells were stimulated in Et-buffer) and incubated in the same buffer at 37 °C with 3 mM of ATP or 5 μM nigericin for different times as indicated in the text. In some experiments, cells were pre-treated with various pharmacological inhibitors or BAPTA-AM 10 min before and during ATP stimulation.

### Liquid chromatography and tandem mass spectrometry (LC-MS/MS)

Fifty micrograms of cell-free supernatant protein were concentrated acetone (6 volume of acetone; overnight at −20 °C), resolved in 4–12% polyacrylamide gels and stained with Comassie blue R-250. Ten gel slices were excised from each lane (for each group) and were subjected to in-gel trypsin digestion as described elsewhere^43^. The tryptic peptides were analyzed by capillary reversed-phase liquid chromatography coupled online with MS/MS. The resulting mass spectra were searched against UniProtKB protein database (453320 sequences, released at June 19, 2012) and *Mus musculus* UniProtKB protein database (69036 sequences, released at May 9, 2011) with the Proteome Discoverer 1.3 software (ThermoScientific). A positive identification was assigned when two or more unique peptides were identified. As criterion we considered proteins that have been identified in at least two of the three independent experiments performed. The mass spectrometry proteomics data have been deposited to the ProteomeXchange Consortium^44^ via the PRIDE partner repository with the dataset identifier PXD001981.

### Antibody protein array

Macrophage supernatants were assayed using the mouse-specific biotin label-based antibody array (RayBio® Biotin Label-based Mouse Antibody Array I, L-Series, RayBiotech, Norcross, USA), that detect 308 different mouse target proteins (Supplementary Table S3).

### Western blots and ELISAs

Cells lysates and precipitated cell-free supernatants were resolved in 4–12% polyacrylamide gels and electrotransferred. Membranes were probed with different antibodies: anti-MMR rat monoclonal (MR5D3, Acris Antibodies), anti-CD14 rat monoclonal (rmC5-3, BD Pharmingen), anti-Cystatin B rat monoclonal (Clone #227818, R&D), anti-Cathepsin B rat monoclonal (Clone #173317, R&D), anti Peptidyl-prolyl cis-trans isomerase A rabbit polyclonal (ab41684, Abcam), anti-Thioredoxin 1 rabbit polyclonal (2298, Cell Signalling), anti-Anxa1 rabbit polyclonal (71–3400, Life Technology), anti-caspase-1 p10 rabbit polyclonal (M-20, sc-514, Santa Cruz), anti-P2X7R rabbit polyclonal (C-terminus, APR-004, Alomone) and anti-NLRP3 mouse monoclonal (Cryo-2 clone, AG-20B-0014, Adipogen). Amounts of IL-1β, TNF-α and CCL2 released to the supernatant were measured by ELISA (R&D Systems).

### Immunofluorescence

Wild-type or *Casp1*^−/−^ *Casp11*^−/−^ BMDM cells were treated as indicated in figures and then stained with Annexin V-FITC for 10 min at RT according to the manufacturer’s instructions (BD Biosciences). Samples were then fixed with 4% formaldehyde in PBS and for cell surface immunostaining, nonspecific binding was blocked by incubation with 1% BSA. Samples were probed with anti-Anxa1 (713400; Life Technology) using Alexa 647 donkey anti-mouse (A-31571; Life technologies) as secondary antibody. Images were acquired with a Nikon Eclipse Ti microscope. Image stacks were deconvolved using NIH ImageJ software with Parallel Iterative Deconvolution plugin, and reconstituted maximum-intensity projections images are shown in the results.

### Lactate dehydrogenase (LDH) determination

Presence of LDH in cell-free supernatants was used as marker for cell death and was measured using the Cytotoxicity Detection kit (Roche, Barcelona, Spain) following the manufacturer’s instructions and read in a Synergy Mx plate reader. Values were expressed as percentage of total cell LDH content.

### Intracellular calcium assay

BMDMs were loaded with the Ca^2+^-sensitive indicator dye Fura-2-AM (4 μM, 40 min, 37 °C). Fluorescence was recorded in a Synergy Mx plate reader (BioTek) for 200 s at 4 s intervals at a λ_exc_ couple 340/380 nm, λ_em_ 510 nm.

### Quantitative reverse transcriptase-PCR analysis

Detailed methods used for qRT-PCR have been described previously^12^.

### Statistical analysis

All data are shown as mean values and error bars represent standard error (s.e.m.) from the number of independent assays indicated in the figure legends. For two-group comparisons, a two-tailed unpaired t-test was used, comparisons of multiple groups were analysed by one-way analysis of variance ANOVA with Bonferroni’s multiple-comparison test using Prism software (Graph-Pad Software, Inc.). *p* value is indicated as ***p < 0.001; **p > 0.001 <0.01; *p > 0.01 <0.05; *p* > 0.05 not significant (ns).

## Additional Information

**How to cite this article**: de Torre-Minguela, C. *et al.* Macrophage activation and polarization modify P2X7 receptor secretome influencing the inflammatory process. *Sci. Rep.* **6**, 22586; doi: 10.1038/srep22586 (2016).

## Supplementary Material

Supplementary Information

## Figures and Tables

**Table 1:** Protein secreted in M1 macrophages upon P2X7R activation.

Protein name	Function	Subcellular localization	SP	Secretion	Unique peptides in LC MS/MS
Transgelin-2 (Q9WVA4)	Protein binding	CP, EVp	−	Unknown	7
Macrophage mannose receptor 1 (Q61830)	Receptor activity	Cell membrane, endosome	+	Non-classical	15
Coactosin-like protein (Q9CQI6)	Protein binding	CP, nucleus membrane, EVp	−	Unknown	3
Tubulin α-1B chain (P05213)	Cytoskeletal organization	CP	−	Unknown	5
Cystatin-B (Q62426)	Protease inhibitor	CP, EVp	−	Non-classical	2
Thioredoxin (P10639)	Redox regulation	CP, mt, nucleus, EVp	−	Non-classical	2
Glutaredoxin-1 (Q9QUH0)	Redox regulation	CP, mt, nucleus, EVp	−	Non-classical	2
Monocyte differentiation antigen CD14 (P10810)	LPS binding	Cell membrane, GPI-anchor	+	Non-classical	5
Elongation factor 1-α 1 (P10126)	Translational elongation factor	CP, nucleus, EVp	−	Unknown	6
GTP-binding nuclear protein Ran (P62827)	Protein binding	CP, nucleus, EVp	−	Unknown	2
SH3 domain-binding glutamic acid-rich-like protein 3 (Q91VW3)	Redox regulation	CP, nucleus, EVp	−	Unknown	2
High Mobility group protein B1 (P63158)	DNA and Protein binding	CP, nucleus	−	Non-classical	2

Relevant proteins secreted after P2X7R activation in M1 macrophages identified by LC-MS/MS. Proteins with Uniprot-UK accession number for *Mus musculus* are denoted in parenthesis. The complete list of proteins identified is described in Supplementary Table S1. Abbreviations and symbols: CP, cytoplasm; EC, extracellular; mt, mitochondria; EVp, extracellular vesicular particles, SP, signal peptide.

**Table 2:** Protein secreted in M2 macrophages upon P2X7R activation.

Protein name	Function	Subcellular localization	SP	Secretion	Unique peptides in LC- MS/MS
Macrophage mannose receptor 1 (Q61830)	Receptor activity	Cell membrane, endosome	+	Non-classical	10
Annexin A1 (P10107)	Ca-dependent phospholipid binding, involve in exocytosis	CP, mt, nucleus, EVp	−	Non-classical	5
Annexin A2 (P07356)	Ca-dependent phospholipid binding, involve in exocytosis	CP, mt, nucleus, EVp	−	Non-classical	3
Annexin A4 (P97429)	Ca-dependent phospholipid binding, involve in exocytosis	CP, EVp	−	Unknown	3
Monocyte differentiation antigen CD14 (P10810)	LPS binding	Cell membrane, GPI-anchor	+	Non-classical	3

Relevant proteins secreted after P2X7R activation in M2 macrophages identified by LC-MS/MS. Proteins with Uniprot-UK accession number for *Mus musculus* are denoted in parenthesis. The complete list of proteins identified is described in Supplementary Table S2. Abbreviations and symbols: CP, cytoplasm; EC, extracellular; mt, mitochondria; EVp, extracellular vesicular particles, SP, signal peptide.

**Table 3:** Protein secreted in M1 macrophages upon P2X7R activation detected by antibodies array.

Protein name	Function	Subcellular localization	SP	Secretion	Fold change intensity
C-C motif chemokine 2 (P10148)	Chemokine	EC	+	Classical	>100*
Artemin (Q9Z0L2)	Co-receptor activity	EC	+	Unknown	>100*
Monocyte differentiation antigen CD14 (P10810)	LPS-binding	Cell membrane (GPI-anchor)	+	Non-Classical	>100*#
C-X-C motif chemokine 9 (P18340)	Chemokine	EC	+	Classical	>100*#
Tumor necrosis factor (P06804)	Cytokine	Cell membrane, EC	+	Non-Classical	>100*#
L-selectin (P18337)	Cell adhesion molecule	Cell membrane	+	Non-Classical	24.25
Interleukin-1β (P10749)	Cytokine	EC	−	Non-classical	9.71
Tyrosine-protein kinase receptor UFO (Q00993)	Receptor Tyrosine kinase	Cell membrane	+	Non-Classical	9.36
Integrin β-2 (P11835)	Receptor activity	Cell membrane	+	Non-Classical	5.57
Kremen protein 2 (Q8K1S7)	Receptor activity	Cell membrane	+	Unknown	5.21
Integrin α-M (P05555)	Receptor activity	Cell membrane	+	Non-Classical	3.30
Membrane frizzled-related protein (Q8K480)	Unknown	Cell membrane	−	Unknown	3.11

Relevant P2X7R-dependent proteins secreted by M1 macrophages identified in antibody array. Proteins with Uniprot-UK accession number for *Mus musculus*. The complete list of proteins obtained is described in Supplementary table S3. Abbreviations and symbols: CP, cytoplasm; EC, extracellular; mt, mitochondria; SP, signal peptide; *no signal in *P2rx7*^−/−^ ; ^#^signal intensity lower of 5% of positive.
