# Supplementary material for: Macrophage activation and polarization modify P2X7 receptor secretome influencing the inflammatory process
Source: Sci Rep. 2016 Mar 3;6:22586. doi: 10.1038/srep22586 (PMC4776275; doi:10.1038/srep22586)
Supplement: Supplementary Information [file srep22586-s1.pdf]

Supplementary Information

Macrophage activation and polarization modify P2X7R secretome influencing the inflammatory process.

Carlos de Torre-Minguela[1], Maria Barberà-Cremades[1], Ana I. Gómez[1], Fátima Martín-Sánchez[1], Pablo Pelegrín[1]

Supplementary Methods

Cells, reagents and buffers

Key reagents and their sources were as follows: *Escherichia coli* LPS serotype 055:B5, ATP, BAPTA-AM, N,N,N',N',-Tetrakis(2-pyridylmethyl) ETH (TPEN), Nigericin sodium salt and Saponin were purchased from Sigma-Aldrich (Madrid, Spain); the cell-permeant irreversible caspase-1 inhibitor IV (Ac-YVAD-AOM) and metaloprotease inhibitors (MMP9 Inhibitor I and GM6001) were from Calbiochem Merck-Millipore (Madrid, Spain); A438079 hydrochloride and Ionomycin free acid were from Tocris (Bristol, UK); and recombinant mouse IL-4 from BD Pharmingen (Madrid, Spain). The composition of the physiological buffer (Et) used in all experiments to stimulate macrophages with ATP or nigericin was (in mM): 147 NaCl, 10 HEPES, 13 D-glucose, 2 KCl, 2 $CaCl_2$, and 1 $MgCl_2$; pH 7.4. In some experiments to block K^+ efflux after P2X7R activation, a high K^+ extracellular solution was used, where 147 mM NaCl was reduced to 2 mM, and 145 mM of KCl was added.

Mice

C57 BL/6 (wild type, WT) mice were purchased from Harlan. P2X7R-deficient mice in C57 BL/6 background (*P2rx7*$^{-/-}$) [1] were purchased from Jackson NLRP3-deficient (*Nlrp3*$^{-/-}$) [2] and Caspase-1 (*Casp1*$^{-/-}$) [3] were in C57 BL/6 background. For all experiments, mice between 8-10 weeks of age bred under SPF conditions were used in accordance with the *Hospital Clínico Universitario Virgen Arrixaca* animal experimentation guidelines, and the Spanish national (RD 1201/2005 and Law 32/2007) and EU (86/609/EEC and 2010/63/EU) legislation.

Differentiation of macrophages from mouse bone marrow precursors

Bone marrow was obtained from leg bones of mice (8–10 weeks of age) euthanized by CO_2 inhalation. Femurs and tibia were removed, and the bone marrow was flushed out and resuspended in DMEM (Lonza, Verviers, Belgium) supplemented with 25% of L929 medium containing macrophage-stimulating factor, 15% FCS, 100 U/ml penicillin-streptomycin, and 2 mM Glutamax, plated onto 150-mm dishes, and cultured at 37°C in the presence of 5% CO_2. After 7 days, the resulting BMDMs were detached with cold PBS, replated into well plates at a confluence of 0.42 x 10^6 cells/cm^2, and used the following day for experiments. In the case of proteomic and antibody array experiments, BMDMs were directly stimulated in 150-mm dishes. The macrophage purity of these preparations was usually >90% and was checked routinely by flow cytometry with the murine macrophage antigen specific Ab F4/80 in a FACScalibur flow cytometer (Beckton-Dickinson Biosciences, Madrid, Spain).

***In vitro* macrophage stimulation**

Macrophage medium was replaced with fresh medium, and cells were primed for 4 h at 37°C with different doses of LPS (as indicated in the figure legends, M1 macrophage polarisation) or with 20 ng/ml of IL-4 (M2 macrophage polarisation) or left in medium alone to obtain resting macrophages. Cells were then rinsed three times with different buffers (Et or Et-high K^+, as indicated in the figure legends, if no indication cells were stimulated in Et-buffer) and incubated in the same buffer at 37°C with 3 mM of ATP or 5 μM nigericin for different times as indicated in the text. In some experiments, cells were pretreated with various pharmacological inhibitors or BAPTA-AM 10 min before and during ATP stimulation To terminate cell stimulation and the release of proteins, the entire volume of extracellular medium was transferred to a tube on ice, and cells were immediately lysed in lysis buffer (50 mM Tris-HCl pH8.0, 150 mM NaCl, 2% Triton X-100) supplemented with 100 μl/ml of protease inhibitor mixture (Sigma) for 30 min on ice and then centrifuged to remove particulate matter. To obtain cell-free supernatants, recovered extracellular medium was clarified by centrifugation at 16,000 x*g* for 30 s to remove floating cells.

One dimension gel electrophoresis (SDS-PAGE)

Protein concentration of cell-free supernatants from stimulated BMDMs was estimated by Bradford assay (Bio-Rad, Hércules, USA) according to manufacturer's instructions. Fifty micrograms of cell-free supernatant protein were precipitated with 6 volume of cold acetone overnight at -20ºC. Protein pellet was then resuspended in Laemmli sample buffer (Serva, Heidelberg, Germany) supplemented with β-mercaptoethanol (4% v/v, Sigma) and resolved in 4–12% precast Criterion polyacrylamide gels (Biorad). Gels were run at 150 V and stained overnight with Comassie blue R-250 (0.1% w/v, Sigma). Ten gel slices were excised from each lane (for each group) and processed for LC-MS/MS. Analysis of the supernatant was performed in triplicate.

Liquid chromatography and tandem mass spectrometry (LC-MS/MS)

The 10 gel slices obtained from the SDS-PAGE gel lanes were subjected to in-gel trypsin digestion. First proteins contained in selected gel bands were reduced and alkylated using dithiothreitol (DTT, 20 mM, Sigma) and iodoacetamide (55 mM, Sigma), respectively, and then digested by trypsin (proteomics grade, Sigma-Aldrich, St Louis, MO, USA) as described elsewhere [4]. The tryptic peptides were analyzed by capillary reversed-phase liquid chromatography coupled online with MS/MS. The column, BioBasic-18, 5 μm particles, 300 Å pore size, 0.18 mm ID-100 mm L (Thermo Scientific, San Jose, USA), was connected to an Surveyor MS Pump Plus (Thermo Scientific, San Jose, CA) and then coupled with an ion trap mass spectrometer (LXQ, Thermo Scientific, San Jose, USA). The flow rate was set at 100 μl/min but split to a flow rate of 2 μl/min. Mobile phase A was 0.1% formic acid / 2% methanol in water and B was 0.1 % formic acid in methanol. The peptide samples were injected and gradient elution was done under the following conditions: 5% B in 3 min; a linear increase of 5% to 70% B in 30 min; 70% for 16 min; 5% B for 43 min. The ion trap MS was operated in a data-dependent MS/MS mode where the 15 most abundant peptide molecular ions in every MS scan were sequentially selected for collision-induced dissociation with a normalized collision energy of 34%. Dynamic exclusion was applied to minimize repeated selection of peptides previously selected for collision-induced dissociation. The capillary temperature and electrospray voltage were set to 200 ºC and 3.5 kV, respectively. The resulting mass spectra were searched against

UniProtKB protein database (453320 sequences, released at June 19, 2012) and *Mus musculus* UniProtKB protein database (69036 sequences, released at May 9, 2011) with the Proteome Discoverer 1.3 software (ThermoScientific). The following search parameters were applied: default charge states of 2+, 3+, and 4+ were used; a maximum of one missed cleavage was allowed with an average peptide mass tolerance of 1.5 Da. A fragment ion search tolerance of 0.8 Da was permitted. Fixed modification on cysteine was carbamidomethylation and a variable modification was oxidation of methionine. All data were searched against a decoy database. The peptide level score cut-off for each of the runs was automatically adjusted to ensure a 1% false discovery rate throughout the experiments. A positive identification was assigned when two or more unique peptides were identified. As criterion we considered proteins that have been identified in at least two of the three independent experiments performed. The mass spectrometry proteomics data have been deposited to the ProteomeXchange Consortium [5] via the PRIDE partner repository with the dataset identifier PXD001981.

Antibody protein array

Macrophage supernatants were assayed using the mouse-specific biotin label-based antibody array (RayBio® Biotin Label-based Mouse Antibody Array I, L-Series, RayBiotech, Norcross, USA), that detect 308 different mouse target proteins, including cytokines, chemokines, adipokines, growth factors, proteases, soluble receptors, soluble adhesion molecules and other proteins (Supplemental Table S3). Briefly, supernatants from LPS/ATP-stimulated wild-type or *P2xr7*$^{-/-}$ BMDMs from 3 independent experiments were pooled and concentrated in PBS pH 8.0 with Ultracel-3K Amicon Ultra (Millipore). Two hundred μg of supernatant protein were biotin-labelled according to manufacturer's instructions. Biotin-labelled supernatant were incubated with the array overnight at 4 °C followed by hybridization with HRP-conjugated streptavidin. Mouse antibody arrays were developed using Pierce ECL2 kit (Pierce, Thermo scientific) and quantified with Typhoon 9410 scanner-ImageQuantTL 7.0 software (GE Healthcare Europe, Barcelona, Spain). The analysis was performed with RayBio® Antibody Array Analysis Tool using a normalization according to positive control densities after background subtraction.

Protein assays by Western blots and ELISAs

Forty milligrams of protein from clarified cells lysates and precipitated cell-free supernatants were resolved in 4–12% precast Criterion polyacrylamide gels (Biorad) and transferred to nitrocelulose membranes (Biorad) by electroblotting. Membranes were incubated overnight at 4 ºC with different antibodies at 1:200 to 1:2000 dilution in blocking buffer: anti-MMR rat monoclonal (MR5D3, Acris Antibodies), anti-CD14 rat monoclonal (rmC5-3, BD Pharmingen), anti-Cystatin B rat monoclonal (Clone #227818) and anti-Cathepsin B rat monoclonal (Clone #173317) were from R&D Systems, anti Peptidyl-prolyl cis-trans isomerase A rabbit polyclonal (ab41684) were from Abcam, anti-Thioredoxin 1 rabbit polyclonal (2298) were from Cell Signaling Technology, anti-Anxa1 rabbit polyclonal (71-3400) were from Life Technologies, anti-caspase-1 p10 rabbit polyclonal (M-20, sc-514) was from Santa Cruz Biotechnology, anti-P2X7R rabbit polyclonal (C-terminus, APR-004) were from Alamone Laboratories and anti-NLRP3 mouse monoclonal (Cryo-2 clone, AG-20B-0014) were from Adipogen. HRP-conjugated secondary anti-rabbit and anti-mouse antibodies were from GE Healthcare, and HRP-conjugated secondary anti-rat antibody was from Jackson ImmunoResearch Laboratories (PA, USA). All HRP-conjugated secondary antibodies were used at 1:5000 dilution and detection using Pierce ECL2 kit and Thyphoon 9410 scanner. Densitometry analyses of membranes were performed using ImageQuant TL (GE Healthcare Europe). The amount of protein secreted was estimated from values of western blot densitometry considering the amount of protein loaded and the total amount of protein obtained from cell extracts. In the supernatant, the proportion of supernatant volume loaded was considered. The total amount of protein was estimated by adding the protein detected in the supernatant and the cellular extract. The ratio of protein secreted in the supernatant was calculated by dividing the protein detected in the supernatant by the total protein.

Amounts of IL-1β, TNF-α and CCL2 released to the supernatant were measured by ELISA (R&D Systems) following the manufacturer's instructions and read in a Synergy Mx plate reader (BioTek, Winooski, VT, USA). Concentration of target cytokine was determined using a standard curve.

Immunofluorescence

Wild-type or Casp1$^{-/-}$Casp11$^{-/-}$ BMDM cells were seeded on poly-L-lysine coated coverslips and incubated overnight before use. Macrophages were primed for 4 h with LPS (1 μg/ml), stimulated for 5 minutes with ATP (3 mM) alone or with saponin 0.01% or EGTA 10 mM and then stained with Annexin V-FITC for 10 min at room temperature according to the manufacturer´s instructions (BD Biosciences). Cells were fixed with 4% formaldehyde in PBS for 15 min at RT, and then washed three times with PBS. For cell surface Anxa1 immunostaining, nonspecific binding was blocked by incubation with 1% bovine serum albumin (Sigma) and cells were incubated with a polyclonal rabbit anti-Anxa1 antibody (1:500 dilution; 713400; Life Technology) for 2h at RT. Cells were washed and then were incubated with Alexa Fluor 647 donkey anti-mouse IgG (1:200 dilution; A-31571; Life technologies) for 1 h at RT. After rinse with PBS, cells were mounted on slides with Prolong Diamond Antifade Mountant with DAPI (Life Technologies). Images were acquired with a Nikon Eclipse Ti microscope equipped with a 20xS Plan Fluor objective (numerical aperture, 0.45), a 40xS Plan Fluor objective (numerical aperture, 0.60) and a 60x Plan Apo Vc objective (numerical aperture, 1.40) and a digital Sight DS-QiMc camera (Nikon) with a Z optical spacing of 0.4 um and 387-nm/447-nm, 472-nm/520-nm and 650-nm/668-nm filter sets (Semrock). Image stacks were deconvolved using NIH ImageJ software with Parallel Iterative Deconvolution plugin, and reconstituted maximum-intensity projections images are shown in the results.

Intracellular calcium assay

BMDMs were plated in 96-well plates with black walls and clear bottoms (Costar Corning Life Sciences, Lowell, MA, USA) and incubated at 37°C for 40 min in loading buffer with 4 μM Fura-2-AM (Invitrogen Life Technologies) supplemented with 0.02% pluronic acid (Merck). At the end of this incubation, Fura-2-AM was removed and replaced with Et buffer. Fluorescence was recorded in a Synergy Mx plate reader (BioTek) for 200 s at 4 s intervals at a λexc couple 340/380 nm, λem 510 nm. ATP and nigericin were automatically injected into the wells at the designated time points. Intracellular calcium level was expressed as the ratio of the emission intensities at 340 and 380 nm, and the value was normalized to the fluorescence at time 0 (F/F_0).

Quantitative reverse transcriptase-PCR analysis

Detailed methods used for qRT-PCR have been described previously [6]. Specific primers were purchased from Qiagen (QuantiTech Primer Assays). For each primer set the efficiency was >95%, and a single product were obtained on melt curve analysis. The presented relative gene expression levels were calculated using the $2^{-\Delta\Delta Ct}$ method normalizing to *Gapdh* expression levels for each treatment and the fold increase in gene expression for LPS or IL-4 treated macrophages was relative to unprimed (resting) macrophages. The fold increase in gene expression for LPS+ATP or IL-4+ATP treated macrophages was relative to LPS or IL-4 treated macrophages respectively.

Supplementary references

1 Solle, M. *et al.* Altered cytokine production in mice lacking P2X(7) receptors. *J Biol Chem* **276**, 125-132 (2001).

2 Martinon, F., Petrilli, V., Mayor, A., Tardivel, A. & Tschopp, J. Gout-associated uric acid crystals activate the NALP3 inflammasome. *Nature* **440**, 237-241 (2006).

3 Kuida, K. *et al.* Altered cytokine export and apoptosis in mice deficient in interleukin-1 beta converting enzyme. *Science* **267**, 2000-2003 (1995).

4 Shevchenko, A., Tomas, H., Havlis, J., Olsen, J. V. & Mann, M. In-gel digestion for mass spectrometric characterization of proteins and proteomes. *Nat Protoc* **1**, 2856-2860 (2006).

5 Vizcaino, J. A. *et al.* ProteomeXchange provides globally coordinated proteomics data submission and dissemination. *Nat Biotechnol* **32**, 223-226 (2014).

6 Lopez-Castejon, G., Baroja-Mazo, A. & Pelegrin, P. Novel macrophage polarization model: from gene expression to identification of new anti-inflammatory molecules. *Cell Mol Life Sci* **68**, 3095-3107 (2011).

Supplementary Figures

Supplementary Figure S1

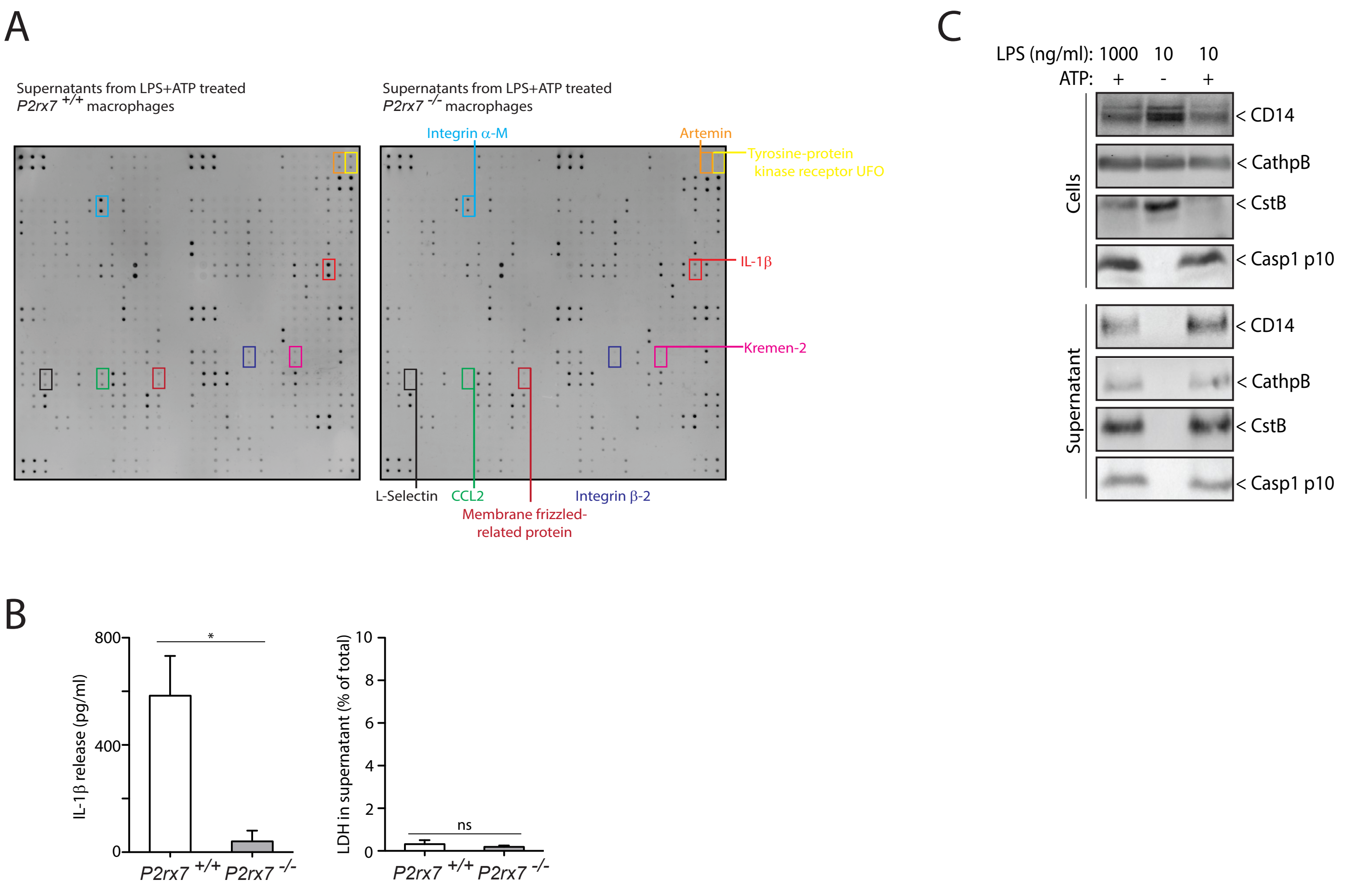

Supplementary Figure S1. Antibody array analysis for P2X7R-dependent secreted cytokines.

A Antibody array analysis of cell-free supernatants from wild-type (*P2rx7*$^{+/+}$) or *P2rx7*$^{-/-}$ mouse bone marrow-derived macrophages (BMDMs) primed for 4 h with LPS (10 ng/ml), followed by stimulation for 20 min with ATP (3 mM). The array was hybridized with a pool of supernatants from n = 3 independent experiments.

B Analysis for IL-1β and LDH in cell-free supernatants from wild-type (*P2rx7*$^{+/+}$) or *P2rx7*$^{-/-}$ mouse bone marrow-derived macrophages (BMDMs) primed for 4 h with LPS (10 ng/ml), followed by stimulation for 20 min with ATP (3 mM); data is presented as mean and s.e.m. of n = 3 independent experiments; *p <0.05; *ns*, p >0.05 (Student's *t*-test).

C Western blot analysis for the release of selected secretome proteins from BMDMs primed for 4 h with different concentrations of LPS as indicated, followed by no stimulation (−) or stimulation (+) for 20 min with ATP (3 mM); Western blots are representative of two experiments.

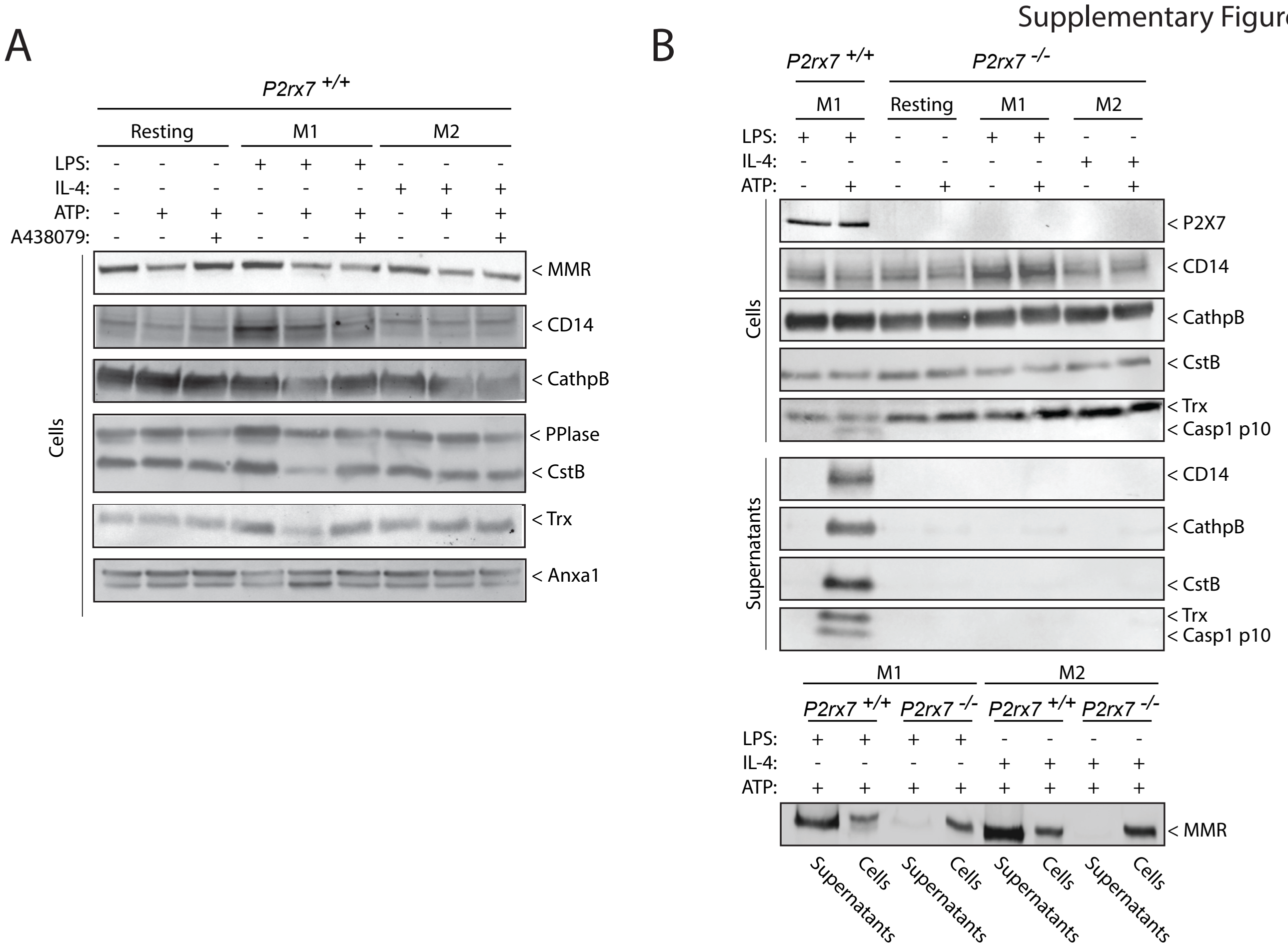

Supplementary Figure S2. Validation of P2X7R-dependent secretome.

A,B Western blot analysis of selected P2X7R secretome proteins in cell lysates (A,B) or cell-free supernatants (B) from wild-type (*P2rx7*$^{+/+}$) (A,B) or P2X7R-deficient (*P2rx7*$^{-/-}$) (B) mouse bone marrow-derived macrophages (BMDMs) unprimed (resting) (−) or primed (+) for 4 h with LPS (1 µg/ml) (M1) or with IL-4 (20 ng/ml) (M2), followed by no stimulation (−) or stimulation (+) of P2X7R for 20 min with ATP (3 mM); when indicated BMDMs were treated 10 min before and during ATP stimulation with the selective P2X7R antagonist A438079 (25 µM). Western blot shown are representative of four independent experiments and correspond to the cell lysates of Western blots shown in Figure 3A.

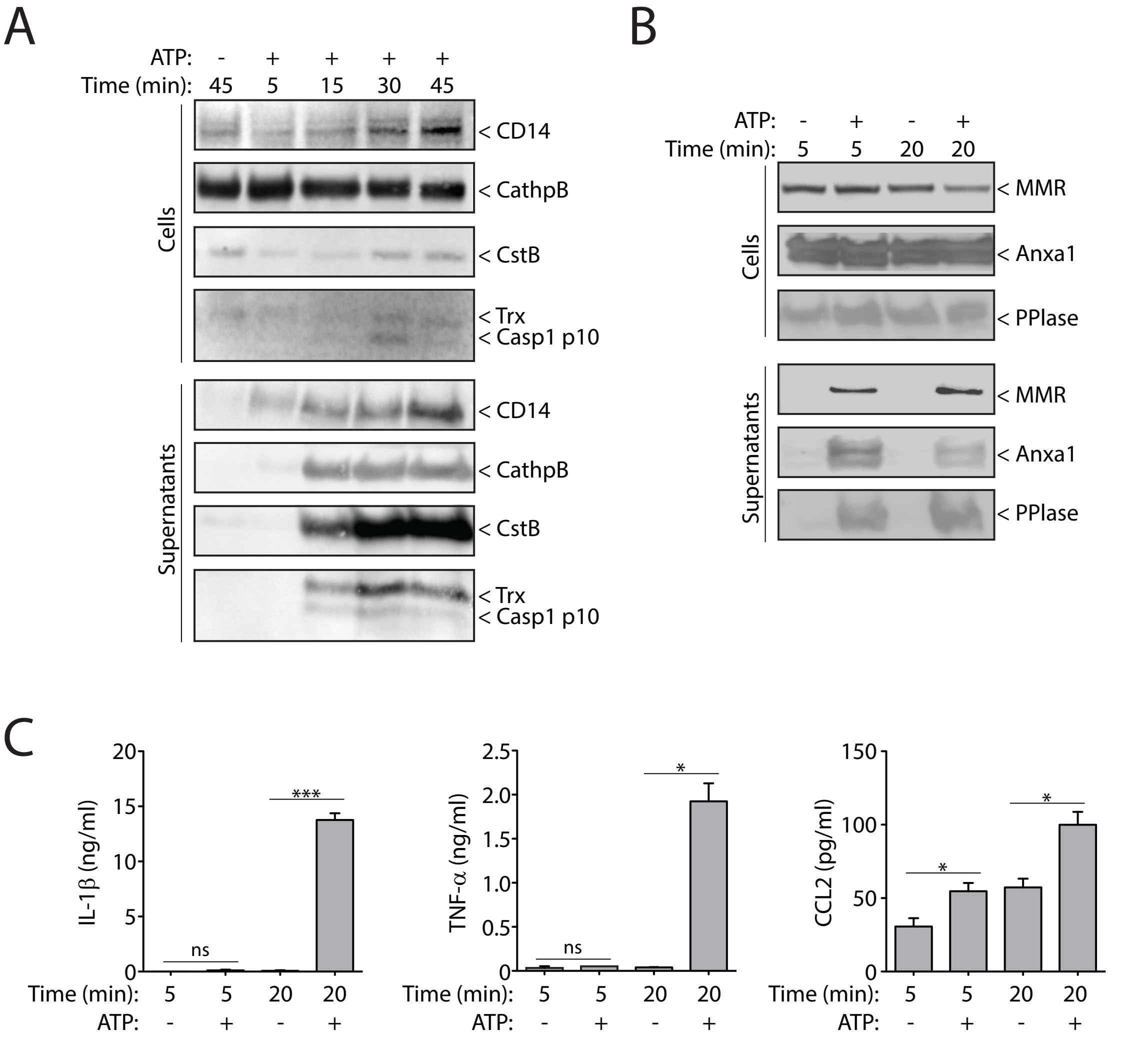

Supplementary Figure S3. P2X7R-dependent secretome kinetics.

A-C Western blots used for quantification (A,B) and ELISAs (C) of selected P2X7R secretome proteins shown in Figure 4A; Western blots are representative of *n* = 1-2 independent experiments (A,B) and ELISA data is presented as mean and s.e.m. of *n* = 2 to 3 independent experiments; ****p* <0.001; ***p* <0.01; **p* <0.05; *ns*, *p* >0.05 (Student's *t*-test).

Supplementary Figure S4

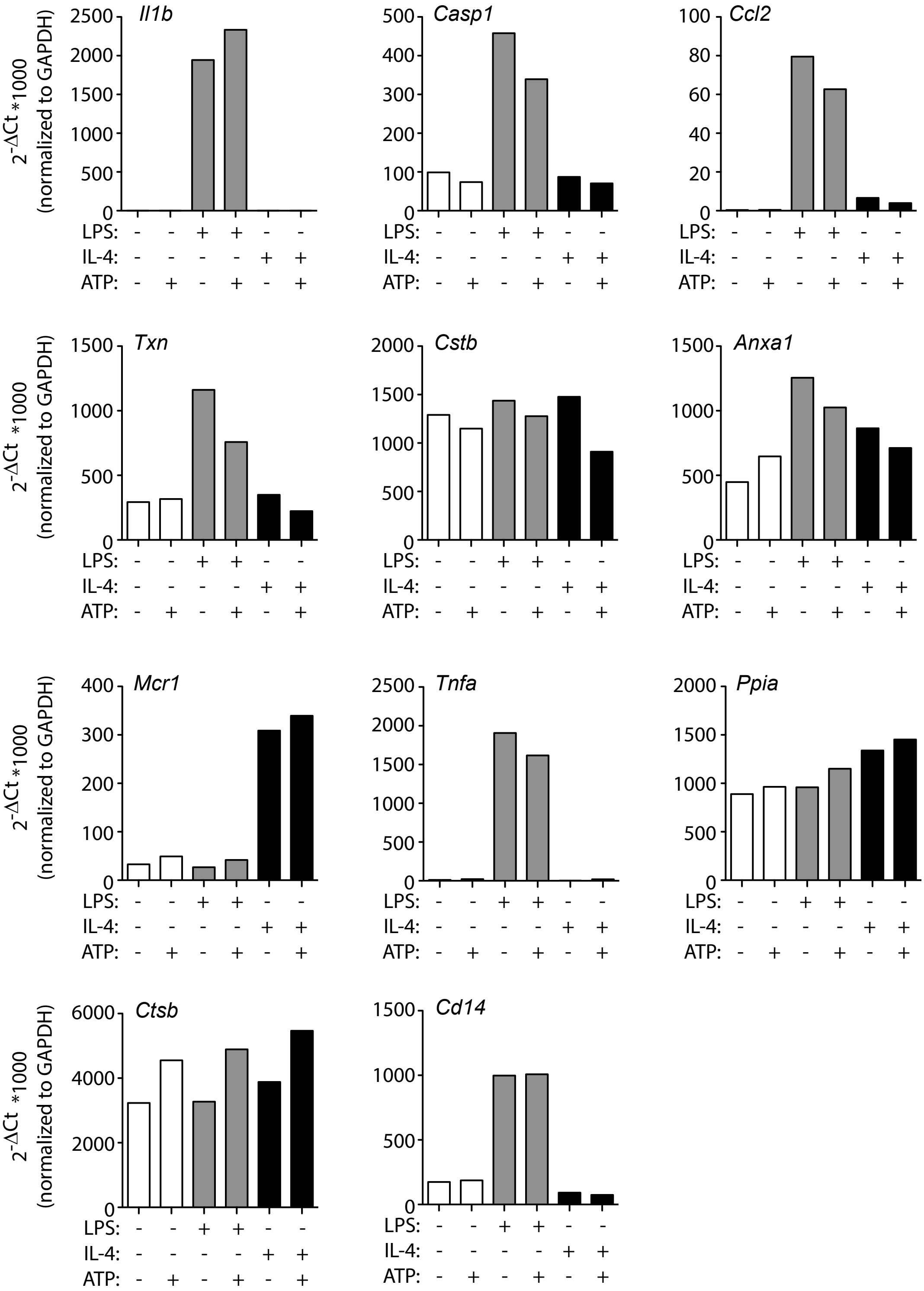

Supplementary Figure S4. Gene expression for P2X7R-dependent secretome proteins.

Relative gene expression for all selected P2X7R secretome proteins shown in Figure 4B determined by quantitative RT-PCR from BMDMs unprimed (−) or primed (+) for 4 h with LPS (1 µg/ml) or with IL-4 (20 ng/ml), followed by no stimulation (−) or stimulation (+) for 20 min with ATP (3 mM); Data is the average of duplicate runs and representative of *n* = 2 independent experiments.

Supplementary Figure S5

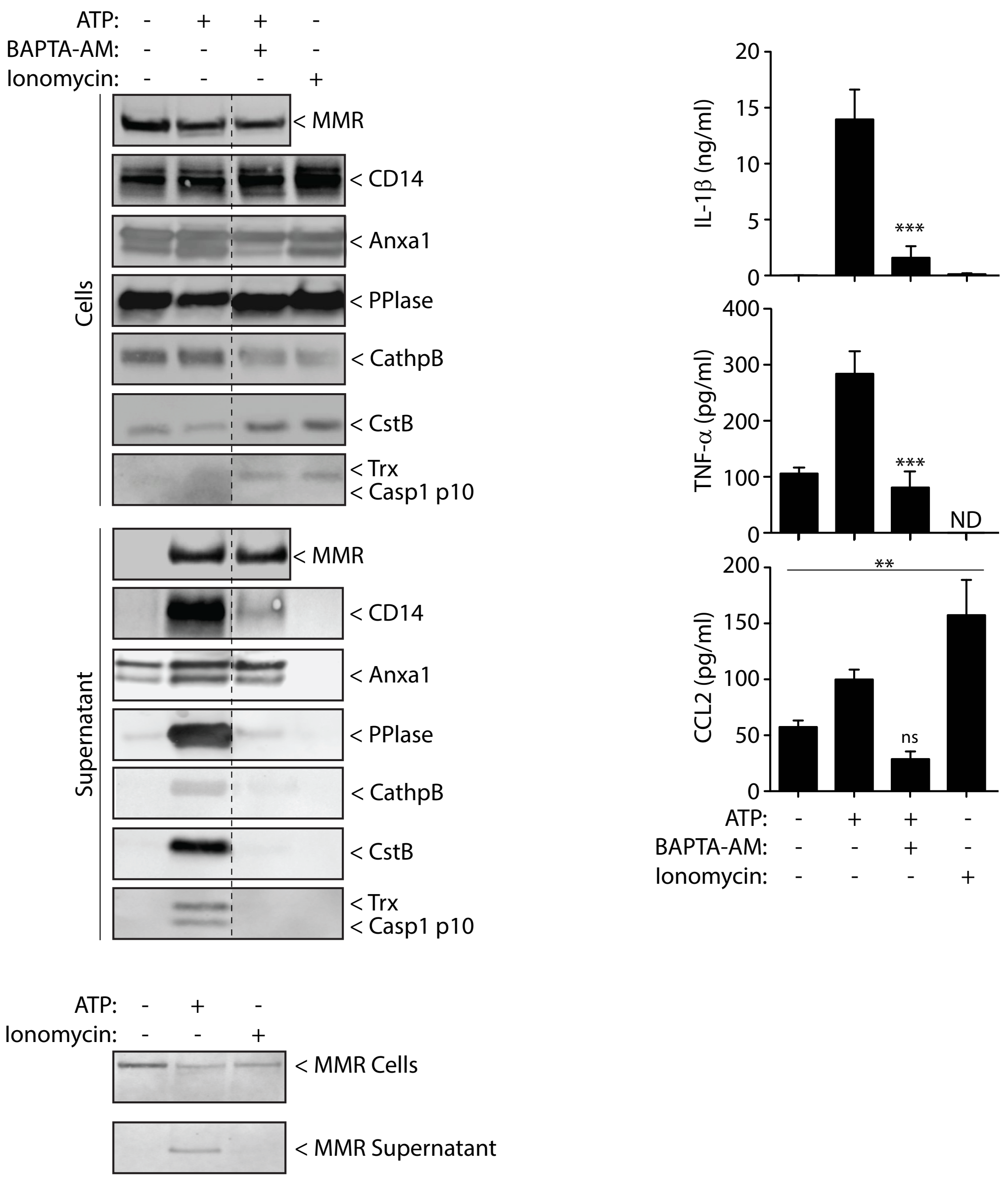

Supplementary Figure S5. Intracellular calcium increase controls part of P2X7R secretome.

Western blots used for quantification and ELISAs of selected P2X7R secretome proteins in cell lysates and cell-free supernatants shown in Figure 6A,B. Mouse bone marrow-derived macrophages (BMDMs) primed for 4 h with LPS (10 ng/ml for CCL2 and TNF-α or 1 μg/ml for the other), followed by no stimulation (−) or stimulation (+) for 20 min with ATP (3 mM) or Ionomycin (5 μM); when indicated BMDMs were treated 10 min before and during ATP stimulation with the cell permeable Ca^{2+}-chelator BAPTA-AM (100 μM). Western blots are representative of *n* = 2 independent experiments and ELISA data is presented as mean and s.e.m. of *n* = 3 independent experiments; ND, not detected. ****p* <0.001; ***p* <0.01; *ns*, *p* >0.05 (Student's *t*-test).

Supplementary Figure S6

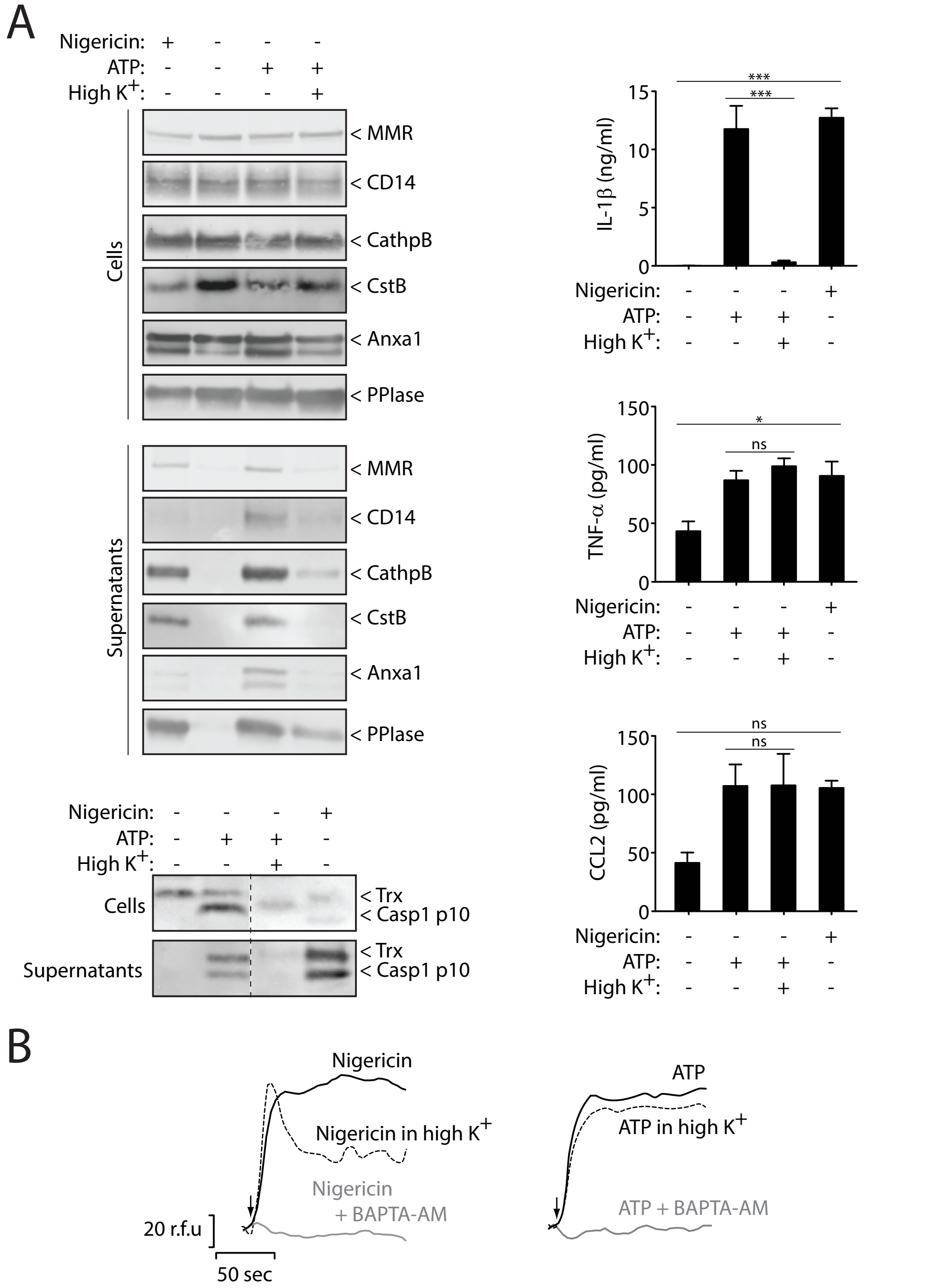

Supplementary Figure S6. Caspase-1 dependent P2X7R secretome.

A Western blots used for quantification and ELISAs of selected P2X7R secretome proteins in cell lysates and cell-free supernatants shown in Figure 7A,B. Mouse bone marrow-derived macrophages (BMDMs) primed for 4 h with LPS (10 ng/ml for CCL2 and TNF-α or 1 μg/ml for the other), followed by no stimulation (−) or stimulation (+) for 20 min with ATP (3 mM) or nigericin (5 μM); when indicated BMDMs were treated with ATP in a buffer with high concentration of K^+ (145 mM). Western blots are representative of n = 2 independent experiments and ELISA data is presented as mean and s.e.m. of n = 3 independent experiments; ***p <0.001; *p <0.05; *ns*, p >0.05 (Student's *t*-test).

B Average trace of 6 independent wells for intracellular calcium rise in BMDMs primed as in A in the presence of absence of the Ca^{2+}-chelator BAPTA-AM (100 μM).

Supplementary Figure S7

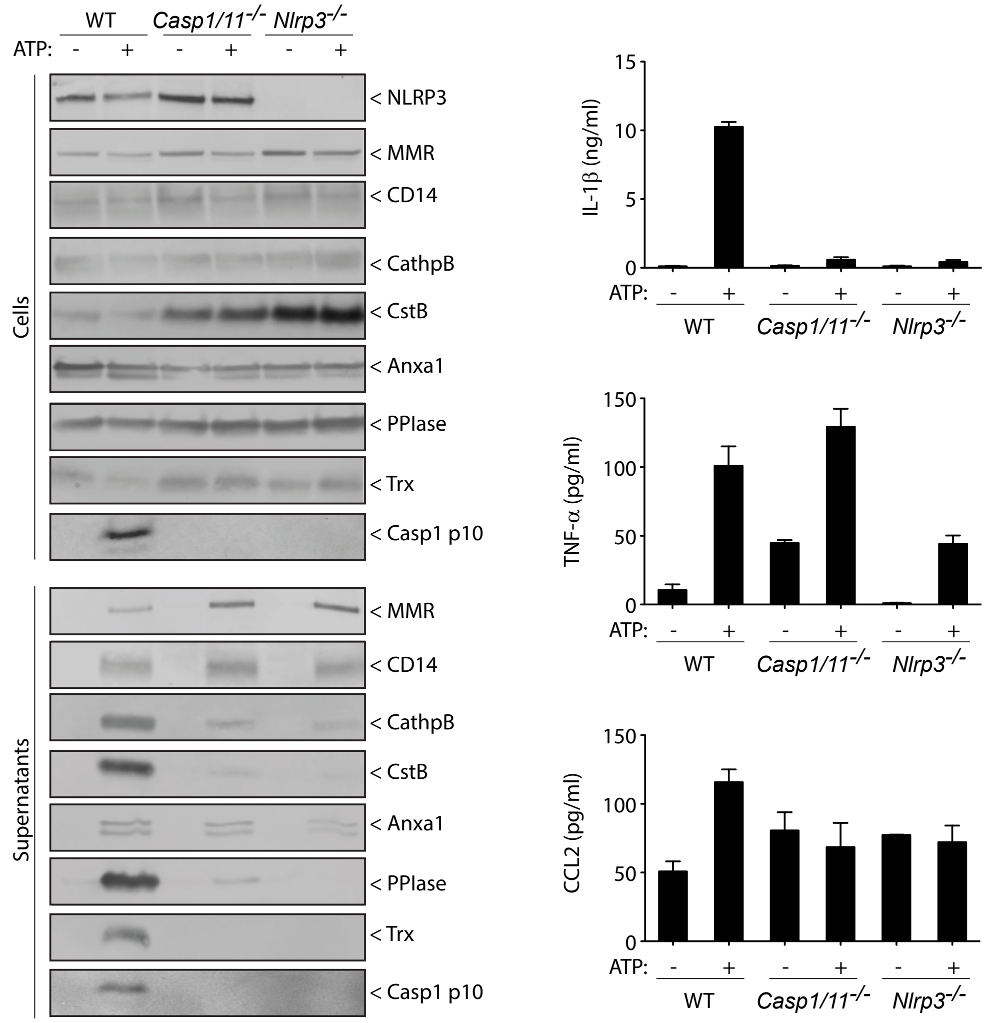

Supplementary Figure S7. Inflammasome and caspase-1 dependent P2X7R secretome.

Western blots used for quantification and ELISAs of selected P2X7R secretome proteins in cell lysates and cell-free supernatants shown in Figure 7C. Mouse bone marrow-derived macrophages (BMDMs) from wild type (WT), *Casp1/11*$^{-/-}$ or *Nlrp3*$^{-/-}$ primed for 4 h with LPS (10 ng/ml for CCL2 and TNF-α or 1 μg/ml for the other), followed by no stimulation (−) or stimulation (+) for 20 min with ATP (3 mM). Western blots are representative of *n* = 2-3 independent experiments and ELISA data is presented as mean and s.e.m. of *n* = 3 independent experiments.

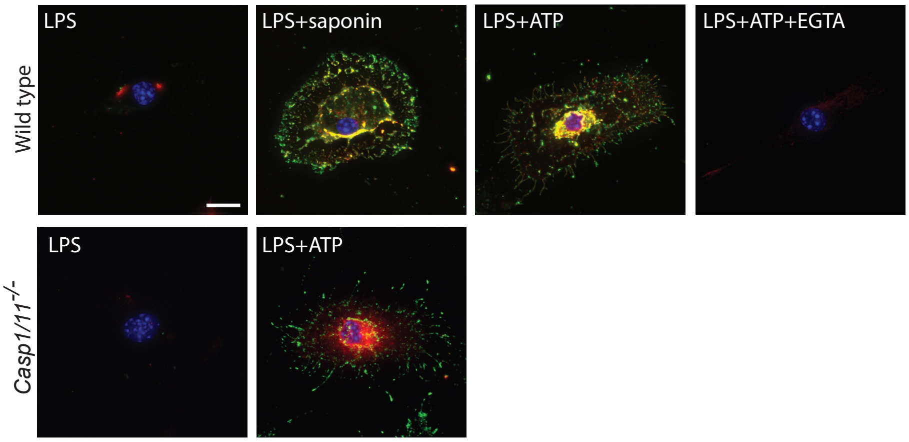

Supplementary Figure S8. P2X7R control the release of metalloprotease.

Representative high intensity projection deconvolved images of mouse bone marrow- derived macrophages (BMDMs) from wild type or *Casp1/11*$^{-/-}$ as indicated primed for 4 h with LPS (1 μg/ml), followed by stimulation or not for 5 min with ATP (3 mM) and stained for phosphatidylserine with annexin V-FITC (green) and for Anxa1 (red) without cell permeabilization and then for nuclei (DAPI, blue); when indicated cells were permeabilised with saponin after fixation (0.01 %), or treated with the Ca^{2+}-chelator EGTA to impair binding of annexins to phosphatidylserine; scale bar = 10 μm.

Supplemental Table S1 .Proteins identified by LC-MS/MS in M1 macrophages

Accesion number (*Mus musculus*)	Protein name	Short name	*P2rx7*$^{-/-}$	*P2rx7*$^{+/+}$	*P2rx7*$^{-/-}$	*P2rx7*$^{+/+}$	Signal peptide	Subcellular Localization [1]	Function [1]	Exosomes[1]	Extracellular vesicle [2]	Phagosome [3]	Score	Unique peptides	Sequence coverage	MW (KDa)
Q9WVA4	Transgelin-2	Tagln2	0	1		***	-	EVp	Protein binding	+	+		323,13	7	37,19%	22,2
Q61830	Macrophage mannose receptor 1	MMR	0	1		***	+	Cell membrane, endosome	Receptor	+	+		359,27	15	12,98%	165
Q9CQI6	Coactosin-like protein	Cotl1	0	1		***	-	CP, nucleus membrane, EVp	Protein binding	+	+		81,33	3	34,51%	15,9
P05213	Tubulin alpha-1B chain	Tuba1b	0	1		**	-	CP	Cytoskeletal organization				185,48	5	18,18%	50,1
Q62426	Cystatin-B	Cstb	0	1		**	-	CP, EVp	Protease inhibitor	+	+		10,63	2	33,67%	11
P10639	Thioredoxin	Trx	0	1		**	-	CP, mt, nucleous, EVp	Redox regulation	+	+		65,47	2	22,86%	11,7
Q9QUH0	Glutaredoxin-1	Glrx	0	1		**	-	CP, mt, nucleous, EVp	Redox regulation	+			23,93	2	19,63%	11,9
P10810	Monocyte differentiation antigen CD14	CD14	0	1		**	+	Cell membrane, GPI-anchor	LPS binding				92,65	5	21,04%	39,2
P10126	Elongation factor 1-alpha 1	EF-1-alpha-1	0	1		**	-	CP, nucleous, EVp	Translational elongation factor	+		+	111,22	6	35,50%	50,1
P62827	GTP-binding nuclear protein Ran	Ran	0	1		**	-	CP, nucleous, EVp	Protein binding	+			16,7	2	31,94%	24,4
Q91VW3	SH3 domain-binding glutamic acid-rich-like protein 3	Sh3bgrl3	0	1		**	-	CP, nucleous, EVp	Redox regulation	+			15,47	2	20,43%	10,5
P63158	High Mobility group protein B1	Hmgb1	0	1		*	-	CP, nucleous,	DNA and Protein binding				13,93	2	12,56%	24,9
Q9DBJ1	*Phosphoglycerate mutase 1*	*Pgam1*	0	1		***	-	CP, EVp	Enzymatic activity	+		+	112,28	6	29,53%	28,8
P17182	Alpha-enolase	Eno1	1	1	*	***	-	CP, EVp	Enzymatic activity	+	+	+	1438,85	18	59,45%	47,1
P10605	Cathepsin B	CathpB	1	1	*	***	+	Lysosome, mt, EVp	Protease	+	+	+	546,24	6	23,30%	37,3
P35700	Peroxiredoxin-1	Prdx1	1	1	*	***	-	CP, mt, EVp	Redox regulation	+	+		193,08	4	23,62%	22,2
P16858	Glyceraldehyde-3-phosphate dehydrogenase	GAPDH	1	1	*	***	-	CP, nucleous, EVp	Enzymatic activity	+	+	+	435,12	6	25,83%	35,8
P16110	Galectin-3	Gal-3	1	1	*	***	-	CP, nucleous, EVp	Lectin activity	+		+	503,04	5	24,24%	27,5
P63101	14-3-3 protein zeta/delta	Ywhaz	1	1	*	***	-	CP, mt, nucleous, EVp	Protein binding	+	+	+	103,04	4	17,55%	27,8
Q99PT1	Rho GDP-dissociation inhibitor 1	Rho GDI 1	1	1	*	***	-	CP, EVp	Protein binding	+		+	371,18	5	30,39%	23,4
P99024	Tubulin beta-5 chain	Tubb5	1	1	*	***	-	CP, nucleous, EVp	Cytoskeletal organization	+	+	+	119,83	9	30,63%	49,6
P18760	Cofilin-1	Cfl1	1	1	*	***	-	CP, EVp	Cytoskeletal organization	+	+		645,57	7	49,40%	18,5
P16045	Galectin-1	Gal-1	1	1	*	***	-	CP, EC, EVp	Apoptosis, differentiation	+	+		398,5	3	28,89%	14,9
Q9DCD0	6-phosphogluconate dehydrogenase, decarboxylating	Pgd	1	1	*	***	-	CP, nucleous, EVp	Enzymatic activity	+	+		370,73	12	29,40%	53,2
P17751	Triosephosphate isomerase	TIM	1	1	*	***	-	CP, EC, nucleous, EVp	Enzymatic activity	+	+		363,58	7	33,33%	26,7
P09411	Phosphoglycerate kinase 1	Pgk1	1	1	*	***	-	CP, EVp	Enzymatic activity	+	+		438,75	11	46,28%	44,5
P60710	Actin, cytoplasmic 1	Actb	1	1	*	***	-	CP, EVp	Cytoskeletal organization	+		+	183,6	4	20,00%	41,7
Q9Z1Q5	Chloride intracellular channel protein 1	NCC27	1	1	*	**	-	CP, EC, mt, nucleous, EVp	Chloride ion channel	+	+		140,84	5	39,83%	27
Q9JII6	Alcohol dehydrogenase [NADP(+)]	Akr1a1	1	1	*	**	-	CP, EC, EVp	Enzymatic activity	+			179,36	4	21,85%	36,6
P52480	Pyruvate kinase isozymes PKM	Pkm	1	1	*	**	-	Nucleous, mt, EVp	Enzymatic activity	+	+		197,2	7	24,29%	57,8
P08905	Lysozyme C-2	Lyz2	1	1	**	***	+	EC	Lysozyme activity	+		+	472,26	2	14,19%	16,7
Q5SX49	Profilin	Pfn1	1	1	**	***	-	CP, nucleous, EVp	Cytoskeletal organization	+	+		420,15	3	35,71%	11,8
P17742	Peptidyl-prolyl cis-trans isomerase A	Pplase A	1	1	**	***	-	CP, EC, nucleous, EVp	Protein folding activity	+	+		597,96	8	63,41%	18

CP, cytoplasm; EC, extracellular; mt, mitochondria; EVp, extracellular vesicular particle
*** In three experiments
** In two experiments
* In one experiments

Selection criteria. Identification in at least two experiments of 6 (3 *P2rx7*$^{+/+}$ and 3 *P2rx7*$^{-/-}$)

[1] UniProtKb database. Reorganizing the protein space at the Universal Protein Resource (UniProt). Nucleic Acids Res. 40: D71-D75 (2012).
[2] Quantitative Proteomics of Extracellular Vesicles Released from Human Monocyte-Derived Macrophages upon β-Glucan Stimulation.Cypryk W, Ohman T, Eskelinen EL, Matikainen S, Nyman TA. J Proteome Res. 2014;13(5):2468-77.
[3]The phagosome proteome: insight into phagosome functions.Garin J, Diez R, Kieffer S, Dermine JF, Duclos S, Gagnon E, Sadoul R, Rondeau C, Desjardins M.J Cell Biol. 2001;152(1):165-80.

Supplemental Table S2 .Proteins identified by LC-MS/MS in M2 macrophages

Accesion number (*Mus musculus*)	Protein name	Short name	*P2rx7*$^{-/-}$	*P2rx7*$^{+/+}$	*P2rx7*$^{-/-}$	*P2rx7*$^{+/+}$	Signal peptide	Subcellular Localization [1]	Function [1]	Exosomes[1]	Extracellular vesicle [2]	Phagosome [3]	Score	Unique peptides	Sequence coverage	MW (KDa)
Q61830	Macrophage mannose receptor 1	MMR	0	1		***	+	Cell membrane, endosome	Receptor	+	+		320,51	10	10,16%	165
P10107	Annexin A1	Anxa1	0	1		**	-	CP, mt, nucleous, EVp	Ca-dependent phospholipid binding, involve in exocytosis	+	+		95,06	5	17,34%	38,7
P07356	Annexin A2	Anxa2	0	1		**	-	CP, cell membrane, EVp	Ca-dependent phospholipid binding, involve in exocytosis	+	+		74,27	3	13,86%	38,7
P97429	Annexin A4	Anxa4	0	1		**	-	CP, EVp	Ca-dependent phospholipid binding, involve in exocytosis	+	+		72,44	3	15,05%	36
P10810	Monocyte differentiation antigen CD14	CD14	0	1		**	+	Cell membrane,GPI-anchor	LPS binding				15,35	3	13,39%	39,2
P08905	*Lysozyme C-2*	*Lyz2*	0	1		**	+	EC	Lysozyme activity	+		+	553,18	2	14,19%	16,7
P17742	*Peptidyl-prolyl cis-trans isomerase A*	*Pplase A*	0	1		**	-	CP, EC, nucleous, EVp	Protein folding activity	+			189,73	3	22,56%	17,9
P63101	14-3-3 protein zeta/delta	Ywhaz	1	1	*	**	-	CP, mt, nucleous, EVp	Protein binding	+	+	+	84,41	3	17,96%	27,8
P16110	Galectin-3	Gal-3	1	1	*	**	-	CP, nucleous, EVp	Lectin activity	+		+	96,13	4	18,56%	27,5
P52480	Pyruvate kinase isozymes PKM	Pkm	1	1	*	**	-	Nucleous, mt, EVp	Enzymatic activity	+	+		30,91	3	6,97%	39,2
P18760	Cofilin-1	Cfl1	1	1	*	**	-	CP, EVp	Cytoskeletal organization	+	+		102,25	3	31,93%	18,5
Q9JII6	Alcohol dehydrogenase [NADP(+)]	Akr1a1	1	1	*	*	-	CP, EC, EVp	Enzymatic activity	+			11,73	2	10,46%	36,6
P62962	Profilin	Pfn1	1	1	*	*	-	CP, nucleous, EVp	Cytoskeletal organization	+	+		44,34	2	23,21%	11,8
Q99PT1	Rho GDP-dissociation inhibitor 1	Rho GDI 1	1	1	*	*	-	CP, EVp	Protein binding	+		+	133,61	2	15,69%	23,4
Q9DBJ1	Phosphoglycerate mutase 1	PGAM-B	1	1	*	*	-	CP, EVp	Enzymatic activity	+		+	11,02	3	19,69%	28,8
Q9Z1Q5	Chloride intracellular channel protein 1	NCC27	1	1	*	*	-	CP, EC, mt, nucleous, EVp	Chloride ion channel	+	+		79,33	2	12,45%	27,1
P17182	Alpha-enolase	Eno1	1	1	**	***	-	CP, EVp	Enzymatic activity	+	+	+	140,14	8	24,88%	47,1
P16858	Glyceraldehyde-3-phosphate dehydrogenase	GAPDH	1	1	**	***	-	CP, nucleous, EVp	Enzymatic activity	+	+	+	110,18	3	13,51%	35,8
P60710	Actin, cytoplasmic 1	Actb	1	1	**	***	-	CP, EVp	Cytoskeletal organization	+	+	+	83,27	2	9,07%	41,7
P10605	Cathepsin B	CathpB	1	1	**	*	+	Lysosome, mt, EVp	Protease	+	+	+	70,12	2	8,85%	37,3
P35700	Peroxiredoxin-1	Prdx1	1	1	***	***	-	CP, mt, EVp	Redox regulation	+	+		333,95	7	45,53%	18,9

CP, cytoplasm; EC, extracellular; mt, mitochondria;
EVp, extracellular vesicular particle
*** In three experiments
** In two experiments
* In one experiments

Selection criteria. Identification in at least two experiments of 6 (3 *P2rx7*$^{+/+}$ and 3 *P2rx7*$^{-/-}$)

[1] UniProtKb database. Reorganizing the protein space at the Universal Protein Resource (UniProt). Nucleic Acids Res. 40: D71-D75 (2012).
2Quantitative Proteomics of Extracellular Vesicles Released from Human Monocyte-Derived Macrophages upon β -Glucan Stimulation.Cypryk W, Ohman T, Eskelinen EL, Matikainen S, Nyman TA. J Proteome Res. 2014;13(5):2468-77.
[3]The phagosome proteome: insight into phagosome functions.Garin J, Diez R, Kieffer S, Dermine JF, Duclos S, Gagnon E, Sadoul R, Rondeau C, Desjardins M.J Cell Biol. 2001;152(1):165-80.

Supplemental Table S3 .Proteins identified by antibodies array in M1 macrophages

Accesion number (*Mus musculus*)	Protein name	Short name	Fold change *P2rx7*$^{+/+}$ / *P2rx7*$^{-/-}$	Signal peptide	Subcellular Localization	Function
P10148	C-C motif chemokine 2	CCL2	>100*	+	EC	Chemokine
Q9Z0L2	Artemin	Artn	>100*	+	EC	Coreceptor activity
P18337	L-selectin	CD62L	24,25	+	Cell membrane	Cell adhesion molecule
P10749	Interleukin 1-beta	IL-1 beta	9,71		EC	Cytokine
Q00993	Tyrosine-protein kinase receptor UFO	Axl	9,36	+	Cell membrane	Receptor Tyrosine kinase. Function in innate immune reponse inhibition
P11835	Integrin beta-2	CD18	5,57	+	Cell membrane	Receptor activity
Q8K1S7	Kremen protein 2	Kremen2	5,21	+	Cell membrane	Receptor activity. Block Wnr/beta-catenin signalling
P05555	Integrin alpha-M	CD11b	3,3	+	Cell membrane	Receptor activity
Q8K480	Membrane frizzled-related protein	Mfrp	3,11		Cell membrane	Unknown
P05366	Serum Amyloid A-1 protein	Saa1	2,56	+	EC	Acute phase protein
P51670	C-C motif chemokine 9	CCL9	2,14	+	EC	Chemokine
Q61271	Activin receptor type-1B	ACTR-IB	2,89	+	Cell membrane	Receptor Ser/Thr kinase
P17125	Transforming growth factor beta-3	TGF-beta-3	2,63	+	EC	Growth factor
O70326	Gremlin	Grem1	2,85	+	EC	Cytokine
Q9JJT2	GDNF family receptor alpha-4	GDNFR-alpha-4	2,69	+	EC, Cell membrane	Coreceptor activity
Q9JKC0	C-C motif chemokine 24	CCL24	2,30	+	EC	Chemokine
P55104	Inhibin beta C chain	Inhbc	2,08	+	EC	Growth factor
P09535	Insulin-like growth factor II	Igf2	2,17	+	EC	Growth factor
Q62401	C-C motif chemokine 12	CCL12	1,72	+	EC	Chemokine
Q03142	Fibroblast growth factor receptor 4	FGFR-4	2,05	+	Cell membrane, endosome	Tyrosine kinase receptor.
P34960	Macrophage metalloelastase	MME	1,63	+	EC	Protease
Q08048	Hepatocyte growth factor	HGF	1,87	+	EC	Growth factor
Q9Z121	C-C motif chemokine 8	CCL8	1,71	+	EC	Chemokine
P97463	Neurturin	Nrtn	>100*#	+	EC	Growth factor
Q64280	Left-right determination factor 1	Lefty1	>100*#	+	EC	Growth factor
Q07104	Growth/differentiation factor 3	GDF-3	>100*#	+	EC	Growth factor
Q8K4C2	Interleukin-17 receptor C	IL-17RC	>100*#	+	Cell membrane	IL-17 receptor
P10810	Monocyte differentiation antigen CD14	CD14	>100*#	+	Cell membrane, GPI-anchor	LPS binding
O55233	Cerberus	Cer1	>100*#	+	EC	Cytokine
P05622	Platelet-derived growth factor receptor beta	PDGFR-beta	>100*#	+	Cell membrane	Receptor Tyrosine kinase.
P18340	C-X-C motif chemokine 9	CXCL9	>100*#	+	EC	Chemokine
P51865	Teratocarcinoma-derived growth factor	Tdgf1	>100*#	+	Cell membrane, GPI-anchor	Growth factor
P41047	Tumor necrosis factor ligand superfamily member 6	CD178	>100*#		EC, cell membrane, EVp	Cytokine
P28862	Stromelysin-1	SL-1	>100*#	+	EC	Protease
P40224	Stromal cell-derived factor 1	SDF-1	>100*#	+	EC	Chemokine
P48356	Leptin receptor	LEP-R	>100*#	+	Cell membrane	Receptor activity
P49764	Placenta growth factor	PlGF	>100*#	+	EC	Growth factor
P15247	Interleukin-9	IL-9	>100*#	+	EC	Cytokine
P11033	Granzyme D	Gzmd	>100*#	+	Cytoplasmic granule	Protease
P26618	Platelet-derived growth factor receptor alpha	PDGFR-alpha	>100*#	+	Cell membrane	Receptor Tyrosine kinase.
P20109	Interleukin-13	IL-13	>100*#	+	EC	Cytokine
Q8K5B1	Interleukin-31 receptor subunit alpha	IL-31RA	>100*#	+	Cell membrane	IL-31 receptor
O88472	Tumor necrosis factor receptor superfamily member 17	CD269	>100*#		Cell membrane	Receptor activity
Q60837	Interleukin-12 receptor subunit beta 1	IL-12R-beta-1	>100*#	+	Cell membrane	IL-12 receptor
Q9JLZ8	Single Ig IL-1 related receptor	Sigirr	>100*#		Cell membrane	Receptor activity
Q9JLL3	Tumor necrosis factor receptor superfamily member 19	Tnfrsf19	>100*#	+	Cell membrane	Receptor activity
Q9EQI5	Thymus Chemokine-1	CXCL7	>100*#			Chemokine
P17515	C-X-C motif chemokine 10	CXCL10	>100*#	+	EC	Chemokine
P20826	Kit ligand	Kitlg	>100*#	+	EC, cell membrane	Growth factor
Q924X1	Epigen	Epgn	>100*#	+	Cell membrane	Growth factor
P48346	Interleukin-15	IL-15	>100*#	+	EC	Cytokine
Q61098	Interleukin-18 receptor 1	IL-18R1	>100*#	+	Cell membrane	IL-18 receptor
P21956	Lactadherin	Mfge8	>100*#	+	EC, cell membrane, EVp	Contributes to phagocytic removal apoptotic cells
Q9JJY9	Interleukin-22	IL-22	>100*#	+	EC	Cytokine
Q62226	Sonic hedgehog protein	SHH	>100*#	+	EC, cell membrane	Protease
Q02858	Angiopoietin-1 receptor	CD202b	>100*#	+	Cell membrane	Receptor Tyrosine kinase.
Q9QUK6	Toll-like receptor 4	CD284	>100*#	+	Cell membrane	Receptor activity
Q9EQC7	Follistatin-related protein 3	Fstl3	>100*#	+	EC	Activin binding
Q61826	Mucosal addressin cell adhesion molecule 1	MAdCAM-1	>100*#	+	Cell membrane	Cell adhesion molecule
P47993	Lymphotactin	Xcl1	>100*#	+	EC	Chemokine
Q9JJN1	Fibroblast growth factor 21	FGF-21	>100*#	+	EC	Growth factor
Q6PHB0	Interleukin-20 receptor subunit alpha	IL-20RA	>100*#	+	Cell membrane	IL-20 receptor
P13366	Granzyme G		>100*#	+	Cytoplasmic granule	Protease
Q61851	Fibroblast growth factor receptor 3	FGFR-3	>100*#	+	Cell membrane	Receptor Tyrosine kinase.
Q9ER04	Transmembrane protease serine 5	Tmprss5	>100*#		EC, cell membrane	Protease
Q5UBV8	Tumor necrosis factor ligand superfamily member 15	Tnfsf15	>100*#		EC, cell membrane	Cytokine
Q62151	Advance glycosylation end product-specific receptor	RAGE	>100*#	+	Cell membrane	Receptor activity
Q8CI19	Platelet-derived growth factor C	PDGF-C	>100*#	+	EC	Growth factor
O88786	Interleukin-13 receptor subunit alpha 2	IL-13RA2	>100*#		EC, cell membrane	Receptor activity
Q64729	TGF-beta receptor type-1	TGFR-1	>100*#	+	Cell membrane	Receptor Ser/Thr kinase
Q9Z0W1	Tumor necrosis factor receptor superfamily member 16	CD271	>100*#	+	Cell membrane	Receptor activity
Q61160	FAS-associated death domain protein	Fadd	>100*#		CP	Protein binding
P06804	Tumor necrosis factor	TNF-a	>100*#		EC, cell membrane	Cytokine
Q9R1V4	Disintegrin and metalloproteinase domain-containing protein 11	ADAM 11	>100*#	+	EC, cell membrane	Protease

* No signal in *P2rx7*$^{-/-}$ samples
\# Signal intensity lower of 5 % of positive

CP, cytoplasm; EC, extracellular; mt, mitochondria; EVp, extracellular vesicular particle
